# Clinical and molecular characteristics of estrogen receptor‐positive ultralow risk breast cancer tumors identified by the 70‐gene signature

**DOI:** 10.1002/ijc.33969

**Published:** 2022-03-07

**Authors:** Annelie Johansson, Nancy Y. Yu, Adina Iftimi, Nicholas P. Tobin, Laura van 't Veer, Bo Nordenskjöld, Christopher C. Benz, Tommy Fornander, Gizeh Perez‐Tenorio, Olle Stål, Laura J. Esserman, Christina Yau, Linda S. Lindström

**Affiliations:** ^1^ Department of Oncology and Pathology Karolinska Institutet and University Hospital Stockholm Sweden; ^2^ Department of Biosciences and Nutrition Karolinska Institutet Stockholm Sweden; ^3^ Department of Laboratory Medicine University of California San Francisco San Francisco California USA; ^4^ Department of Pathology University of California San Francisco San Francisco California USA; ^5^ Department of Biomedical and Clinical Sciences and Department of Oncology Linköping University Linköping Sweden; ^6^ Department of Medicine University of California San Francisco San Francisco California USA; ^7^ Buck Institute for Research on Aging Novato California USA; ^8^ Department of Surgery University of California San Francisco San Francisco California USA

**Keywords:** 70‐gene signature, breast cancer, gene expression, long‐term survival, prognosis, ultralow risk

## Abstract

The metastatic potential of estrogen receptor (ER)‐positive breast cancers is heterogeneous and distant recurrences occur months to decades after primary diagnosis. We have previously shown that patients with tumors classified as ultralow risk by the 70‐gene signature have a minimal long‐term risk of fatal breast cancer. Here, we evaluate the previously unexplored underlying clinical and molecular characteristics of ultralow risk tumors in 538 ER‐positive patients from the Stockholm tamoxifen randomized trial (STO‐3). Out of the 98 ultralow risk tumors, 89% were luminal A molecular subtype, whereas 26% of luminal A tumors were of ultralow risk. Compared to other ER‐positive tumors, ultralow risk tumors were significantly (Fisher's test, *P* < .05) more likely to be of smaller tumor size, lower grade, progesterone receptor (PR)‐positive, human epidermal growth factor 2 (HER2)‐negative and have low Ki‐67 levels (proliferation‐marker). Moreover, ultralow risk tumors showed significantly lower expression scores of multi‐gene modules associated with the AKT/mTOR‐pathway, proliferation (AURKA), HER2/*ERBB2‐*signaling, IGF1‐pathway, PTEN‐loss and immune response (IMMUNE1 and IMMUNE2) and higher expression scores of the PIK3CA‐mutation‐associated module. Furthermore, 706 genes were significantly (FDR < 0.001) differentially expressed in ultralow risk tumors, including lower expression of genes involved in immune response, PI3K/Akt/mTOR‐pathway, histones, cell cycle, DNA repair, apoptosis and higher expression of genes coding for epithelial‐to‐mesenchymal transition and homeobox proteins, among others. In conclusion, ultralow risk tumors, associated with minimal long‐term risk of fatal disease, differ from other ER‐positive tumors, including luminal A molecular subtype tumors. Identification of these characteristics is important to improve our prediction of nonfatal vs fatal breast cancer.

## Graphical abstract

What's new?

The metastatic potential of estrogen receptor (ER)‐positive breast cancers is heterogeneous, and distant recurrences may occur months to decades after primary diagnosis. However, the long‐term risk of metastatic disease in breast cancer remains largely unexplored. Using a previously‐established 70‐gene signature to identify breast cancer patients with minimal long‐term risk of fatal disease, here the authors show that ultralow‐risk tumors differ from other ER‐positive breast cancer tumors, including luminal molecular subtype tumors. Differences were found in both clinical breast cancer markers and molecular features. These results are important for the characterization and prediction of non‐fatal vs fatal breast cancer.

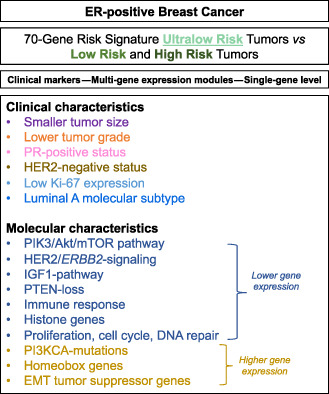

**Abbreviations**
ER: estrogen receptor
FDR: false‐discovery rate
FFPE: formalin‐fixed paraffin‐embedded
HER2: human epidermal growth factor receptor 2
IHC: immunohistochemistry
PR: progesterone receptor

## 1 INTRODUCTION

Estrogen receptor (ER)‐positive breast cancer patients have a steady long‐term risk of fatal disease, and distant metastatic recurrence can occur anywhere between a few months to several decades after primary diagnosis.^1^, ^2^, ^3^, ^4^, ^5^, ^6^ Mammographic screening enables detection of early breast cancer and has reduced the disease mortality, but can introduce overdiagnosis of tumors that might never have come to clinical attention.^7^, ^8^ Adding molecular risk prediction tools to standard clinical breast cancer markers may improve risk assessment and reduce overtreatment in patients with low risk of metastatic disease.^9^ However, to confidently offer less aggressive treatment to patients, a better understanding of the long‐term risk of metastatic disease in breast cancer is needed.

The 70‐gene signature (MammaPrint) was originally designed to identify breast cancer patients with high or low risk of early relapse within 5 years after primary diagnosis to identify which patients require adjuvant therapy.^10^ The signature was developed in lymph node‐negative patients under the age of 55, but has shown to also be prognostic in lymph node‐positive and older patients, and up to 25 years after primary diagnosis.^11^, ^12^, ^13^ Genes included in the signature and upregulated in high‐risk patient tumor samples have been shown to be associated with the cell cycle, invasion and metastasis and angiogenesis.^10^ Clinical trials have validated that ER‐positive patients of otherwise high clinical risk, but classified as low risk by the 70‐gene signature, may not benefit from adjuvant chemotherapy,^14^ and this molecular risk prediction tool is implemented in several breast cancer treatment guidelines.^15^, ^16^, ^17^ Moreover, the additional information from the 70‐gene signature has been shown to improve clinicians' confidence in their treatment recommendations.^18^, ^19^, ^20^ Furthermore, we have demonstrated that the “ultralow risk” threshold derived from the 70‐gene signature identifies patients with a very low long‐term risk of fatal breast cancer. The 20‐year disease‐specific survival was 97% and 94% for ER‐positive lymph‐node negative patients randomized to tamoxifen vs no adjuvant therapy, respectively.^9^ Consequently, it is important to understand the underlying characteristics of ultralow risk breast cancer tumors, given that the long‐term risk in breast cancer is largely unexplored.

Therefore, here we aimed to assess the clinical and molecular characteristics of ultralow risk tumors in ER‐positive lymph‐node negative postmenopausal breast cancer patients from the Stockholm tamoxifen randomized trial (STO‐3). Ultralow risk tumors were compared to other ER‐positive tumors, including PAM50 molecular subtype luminal A and B tumors, regarding the clinically used breast cancer markers, as well as the expression scores of 19 multigene modules representative of specific biological processes and pathways. Furthermore, differentially expressed genes on the single‐gene level in ultralow risk tumors vs other ER‐positive tumors were identified and categorized by their associated Hallmark gene set to better understand the molecular characteristics of ER‐positive tumors with very low long‐term risk of fatal disease.

## 2 MATERIALS AND METHODS

### 2.1 Patients

The Stockholm breast cancer study group conducted randomized trials 1976 to 1990 in lymph‐node negative postmenopausal patients with tumors ≤30 mm in diameter.^21^, ^22^ The Stockholm tamoxifen trial (STO‐3) enrolled 1780 patients that were randomized to 2 years of adjuvant tamoxifen (40 mg daily) vs no adjuvant treatment.^21^, ^22^ In 1983, patients who re‐consented and were recurrence‐free after 2 years of tamoxifen treatment were randomized to 3 additional years of tamoxifen. As a result, patients in the tamoxifen randomization arm were treated with tamoxifen for 2 or 5 years. No significant differences in survival in the comparison of 2 vs 5 years of tamoxifen have been observed in the STO‐3 trial.^21^

**FIGURE 1:**
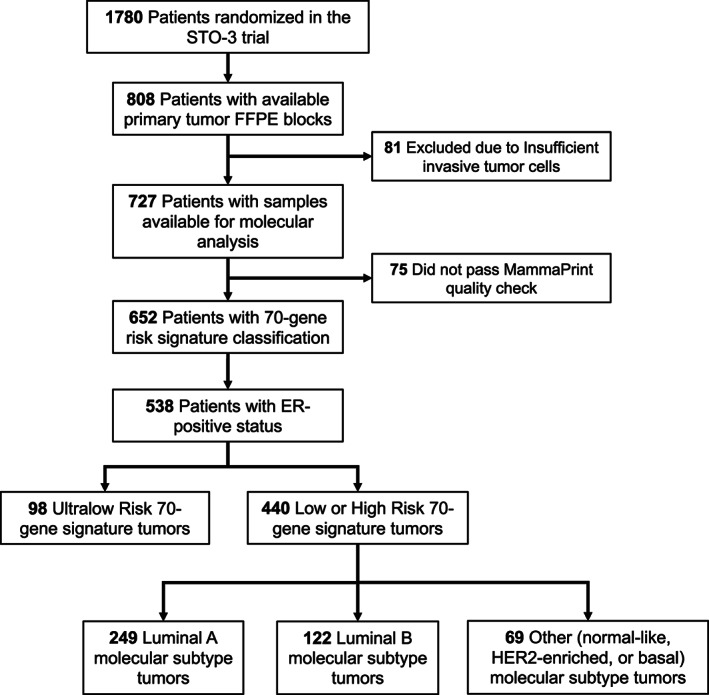
Participant flowchart of the Stockholm tamoxifen randomized trial (STO‐3)

Molecular analysis was possible for 808 patients with available formalin‐fixed paraffin‐embedded (FFPE) tissue blocks from the primary breast cancer tumor.^23^ The 808 patient subset was well balanced to the original STO‐3 trial cohort with regards to tumor characteristics.^23^ Eighty‐one patients were excluded from analysis due to insufficient invasive tumor cells, leaving 727 samples available for further analysis (Figure 1).

### 2.2 Immunohistochemistry

Immunohistochemistry (IHC) analysis of ER, progesterone receptor (PR), human epidermal growth factor 2 (HER2) and proliferation marker Ki‐67 were performed 2014.^2^ The annotation of whole‐tumor sections (5 μm) from FFPE tumor blocks was conducted in a random order at a single medical laboratory (University of California Davis Medical Center, UCDMC) following standard recommended procedures. The slides were stained using DAKO Link48 Autostainer with antibodies for ER (SP1; Spring Bioscience M301), PR (PgR 636; DAKO IR068), HER2 (HercepTest; DAKO SK001) and Ki‐67 (MIB‐1; DAKO M7240). The percentage of cancer cells positive for ER, PR, HER2 and Ki‐67 was scored by breast cancer pathologists.^2^ ER‐ and PR‐positivity was defined by a threshold of 10% or greater, according to the Swedish National Guidelines,^24^ HER2‐positivity as intensity 3+ by IHC and Ki‐67 was categorized as low (<15%) and medium/high (≥15%).

### 2.3 Tumor grade

Tumor grade was retrospectively assessed by one pathologist according to the Nottingham Histologic Score system (Elston grade).^23^

### 2.4 Agilent microarray gene expression profiling

Agilent microarray profiling was performed in 2014.^2^ Gene expression data were independently generated using custom‐designed arrays, Agilent Technologies (Santa Clara, CA), containing approximately 32.1K probes, representing approximately 21.5K unique genes from FFPE breast cancer tumor tissue. Out of 727, 652 breast cancer tumors passed the RNA quality check according to the diagnostic quality model and were used in the analysis, of which 538 were ER‐positive (Figure 1).

### 2.5 70‐Gene signature risk classification

The 70‐gene signature (MammaPrint) was used to classify primary tumors into “high risk”, “low risk” or “ultralow risk.” The molecular risk prediction tool was originally designed to identify patients with low or high risk of early relapse,^10^, ^11^, ^12^, ^13^ and the ultralow risk threshold was added to identify patients with indolent tumors associated with minimal long‐term risk of fatal disease.^9^ From the primary tumor microarray gene expression data, the 70‐gene signature risk classification was performed according to standard protocols as previously described, including the use of 465 normalization genes and over 250 probes for hybridization and printing quality control.^11^, ^25^ Patient tumor samples were classified into “high risk” (<0), “low (but not ultralow) risk” (>0, <0.355) and “ultralow risk” (≥0.355) using thresholds previously developed.^9^, ^11^

### 2.6 Molecular subtyping

Tumors were assigned to one of five molecular subtypes: luminal A, luminal B, HER2‐enriched, basal or normal‐like, by the PAM50 classification,^26^ see details in Supporting Information Materials and Methods.

### 2.7 Multigene modules expression scores

The expression scores of 19 multigene modules, proxy‐signatures for activation of biological processes or pathways and associated with clinical outcome, were analyzed in the study.^27^, ^28^, ^29^ Each module comprises genes that are positively or negatively associated with its biological process/pathway. Continuous expression scores for each multigene module were calculated for all 652 STO‐3 patients with gene expression data using R package Genefu version 2.8.0.^30^ The continuous multigene expression scores were categorized to tertiles, which were then converted to two values: the most aggressive tertile (low or high expression scores suggested to be associated with worse prognosis, see details in Supporting Information Materials and Methods) vs the two less aggressive tertiles combined.^27^ For example, the highest expression tertile for the proliferation‐marker AURKA, associated with higher proliferation and clinical aggressiveness, was compared to the two lower expression tertiles. The multigene module expression scores in tumors classified as “ultralow risk” according to the 70‐gene signature were compared to three other groups of tumors classified as either “low risk” or “high risk”: (a) all other ER‐positive tumors, (b) ER‐positive luminal A molecular subtype tumors and (c) ER‐positive luminal B molecular subtype tumors.

### 2.8 Differentially expressed genes

On a single‐gene level, an exploratory analysis of genes differentially expressed in ultralow risk tumors compared to all other ER‐positive tumors of low or high risk was conducted. Prior to this, probes from the gene expression microarray data were filtered out if the median expression value was among the bottom 5%, if the variance was among the 25% lowest, if containing any missing values, or if the probe was not annotated to any gene. The differentially expressed genes were categorized by Hallmark gene sets (version 6.2)^31^ and Gene Ontology biological processes (C5 collection version 6.2),^32^ which were combined into 27 Hallmark gene sets, see Table S1 and Supporting Information Materials and Methods. A heatmap for the differentially expressed genes was produced using Z‐scores of normalized gene expressions of all 538 ER‐positive tumors, ordered by the 70‐gene signature classification, molecular subtype, tumor grade, tumor size, PR status, HER2 status and Ki‐67 status.

### 2.9 Gene set enrichment analysis

Using the 27 Hallmark gene sets, a gene set enrichment analysis (GSEA) was performed to identify which gene sets are significantly enriched of genes differentially expressed in ultralow risk tumors compared to other ER‐positive tumors of low or high risk. The GSEA calculates enrichment scores (ES) for each gene set to explore if genes belonging to the gene set tend to occur at the top (or bottom) of a specific preranked gene list.^33^ Genes were ranked using the *t*‐statistics output from the analysis of differentially expressed genes. A false‐discovery rate (FDR) of 5% was used to adjust for multiple testing.

### 2.10 Statistical analysis

Given that all ultralow risk tumors were ER‐positive, only ER‐positive patients were included in our study (n = 538; Figure 1). Fisher's exact test was used to compare ultralow risk tumors to other ER‐positive tumors by the clinically used breast cancer markers (ie, tumor size, tumor grade, PR, HER2 and Ki‐67) and the 19 multigene modules expression scores. A *P*‐value less than .05 was considered statistically significant. The analysis to identify differentially expressed genes was conducted using R package OCplus^34^ with t‐statistics and FDR cutoff of 0.001.

All data preparation and analysis were done using R version 3.5.2 and SAS software version 9.4. All statistical tests were two‐sided.

## 3 RESULTS

A total of 652 patients in the STO‐3 trial with 70‐gene signature risk classification were available for analysis. The 70‐gene signature classified 15% (n = 98) of the tumors as of ultralow risk (Figure 1). Given that all ultralow risk tumors were ER‐positive as determined by IHC, the analyses were focused on the 538 patients with ER‐positive breast cancer tumors only (Figure 1). In Table 1, patient and tumor characteristics are presented for ultralow risk tumors, all other ER‐positive tumors (of low or high risk) and ER‐positive luminal A and B molecular subtype tumors (of low or high risk). Noteworthy, a majority (n = 87 out of 98, 89%) of the ultralow risk tumors were of luminal A molecular subtype (Table S2). On the other hand, only 26% (87 out of 336) of the luminal A molecular subtype tumors were classified as ultralow risk. Moreover, four ultralow risk tumors were of luminal B subtype, seven normal‐like and none of HER2‐enriched or basal molecular subtype.

**TABLE 1:** Primary patient and tumor characteristics of ER‐positive patients in the STO‐3 trial

Primary patient and tumor characteristics	Ultralow risk tumors (n = 98)	ER‐positive tumors of low or high risk					
		All other ER‐positive tumors (n = 440)		Luminal A tumors (n = 249)		Luminal B tumors (n = 122)	
	No (%)	No (%)	*P* ^b^	No (%)	*P* ^b^	No (%)	*P* ^b^
Tumor size							
pT ≤ 20 mm	89 (90.8)	348 (80.2)	**.013**	205 (83.7)	.123	83 (68.0)	**<.001**
pT > 20 mm	9 (9.2)	86 (19.8)		40 (16.3)		39 (32.0)	
Unknown	0 (—)	6 (—)		4 (—)		0 (—)	
Tumor grade							
1	39 (39.8)	77 (17.8)	**<.001**	58 (23.5)	**<.001**	5 (4.2)	**<.001**
2	59 (60.2)	279 (64.6)		172 (69.6)		74 (62.2)	
3	0 (0.0)	76 (17.6)		17 (6.9)		40 (33.6)	
Unknown	0 (—)	8 (—)		2 (—)		3 (—)	
PR status^a^							
Positive	82 (84.5)	285 (66.0)	**<.001**	174 (71.6)	**.012**	74 (61.2)	**<.001**
Negative	15 (15.5)	147 (34.0)		69 (28.4)		47 (38.8)	
Unknown	1 (—)	8 (—)		6 (—)		1 (—)	
HER2 status^a^							
Negative	98 (100.0)	415 (94.5)	**.012**	246 (98.8)	.562	117 (95.9)	.067
Positive	0 (0.0)	24 (5.5)		3 (1.2)		5 (4.1)	
Unknown	0 (—)	1 (—)		0 (—)		0 (—)	
Ki‐67 status^a^							
Low	89 (95.7)	306 (73.0)	**<.001**	196 (82.7)	**.001**	69 (58.5)	**<.001**
Medium/High	4 (4.3)	113 (27.0)		41 (17.3)		49 (41.5)	
Unknown	5 (—)	21 (—)		12 (—)		4 (—)	

^a^ PR‐positivity was defined as ≥10%, HER2‐positivity as intensity 3+ and Ki‐67 was categorized as low (<15%) and medium/high (≥15%).

^b^ Fisher's exact test, using ultralow risk tumors as reference. Significant *P* values (*P* < .05) are marked in bold.

### 3.1 Clinical characteristics of ultralow risk breast cancer tumors

The ultralow risk tumors were significantly (*P* < .05) more likely to be of a smaller (pT ≤ 20 mm) tumor size, except in the comparison of luminal A tumors and of lower tumor grade, as compared to the other groups (Table 1). Furthermore, ultralow risk tumors differed from other ER‐positive tumors in the IHC markers by being significantly more likely to be PR‐positive, HER2‐negative and Ki‐67‐low (<15%; Table 1). Note, however, that all ultralow risk tumors were of grade 1‐2 and HER2‐negative, and that HER2‐status did not significantly differentiate ultralow risk tumors to luminal A or B molecular subtype tumors.

### 3.2 Multigene module expression scores in ultralow risk breast cancer tumors

**FIGURE 2:**
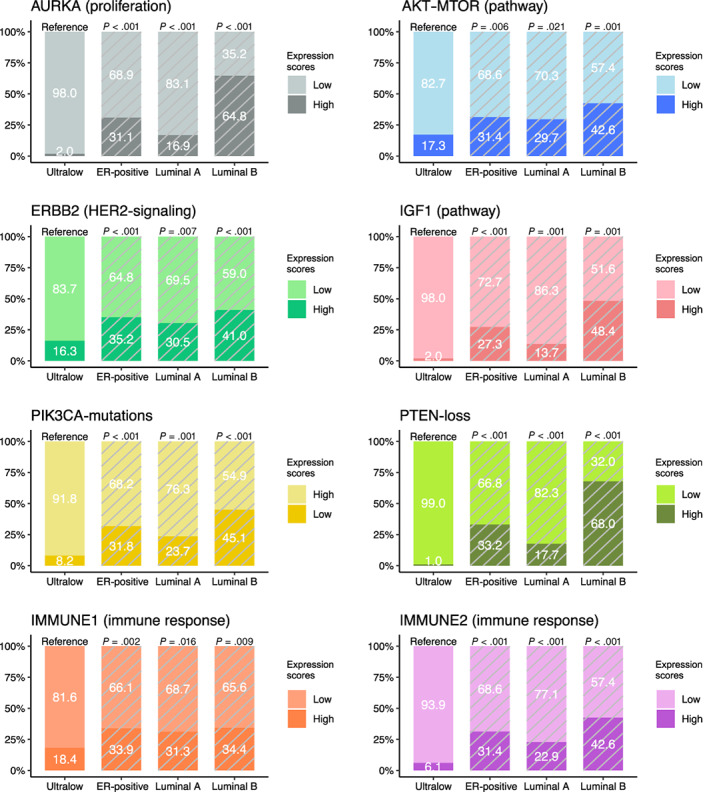
Multigene module expression scores in ultralow risk breast cancer tumors. Bar plot of the eight multigene modules with significantly different expression scores in ultralow risk breast cancer tumors compared to all other ER‐positive tumors (of low or high risk), and ER‐positive luminal A and luminal B molecular subtype tumors (of low or high risk). The plot shows the percentages of tumors with low or high expression scores. The modules included are proliferation‐marker AURKA, AKT/mTOR‐pathway, HER2/*ERBB2*‐signaling, IGF1‐pathway, PIK3CA‐mutation‐associated module, PTEN‐loss and immune response modules IMMUNE1 and IMMUNE2. Darker colors represent the category of module‐expression scores suggested to be associated with worse prognosis (ie, high AURKA expression scores, but low PIK3CA‐mutation expression scores) and diagonal lines indicates significant differences from ultralow risk tumors (reference) by Fisher's exact test (*P* < .05)

In order to further understand the molecular characteristics of ultralow risk tumors, the expression scores of 19 multigene modules (proxy‐signatures associated with different biological pathways/processes and clinical outcome) in the ultralow risk tumors were compared to all other ER‐positive tumors (of low or high risk), and ER‐positive luminal A and B molecular subtype tumors (of low or high risk). Eight multigene modules showed significantly (*P* < .05) different expression scores in ultralow risk tumors (Figure 2). Lower expression scores of the proliferation‐marker AURKA, AKT/mTOR‐pathway, HER2/*ERBB2*‐signaling, IGF1‐pathway, PTEN‐loss and the immune response‐modules IMMUNE1 and IMMUNE2 were observed for the ultralow risk tumors, and higher expression scores were observed for the PIK3CA‐mutation‐associated module (Figure 2). Moreover, compared to all other ER‐positive tumors, but not the luminal molecular subtypes, ultralow risk tumors showed significantly higher expression scores of the *ESR1*‐module (Table S3). Furthermore, lower expression scores of the pathway‐associated gene‐modules MYC and E2F3 were observed in ultralow risk tumors compared to all other ER‐positive tumors and luminal B molecular subtype tumors (Table S3). Other multigene modules differentiating ultralow risk tumors from luminal B molecular subtype tumors included lower expression scores of CASP3, associated with apoptosis and higher expression scores of MAPK, STROMA1 and STROMA2, associated with its pathway, tumor invasion/metastasis and the stromal environment, respectively.

### 3.3 Differentially expressed genes in ultralow risk breast cancer tumors

To further understand the molecular characteristics of ultralow risk tumors, an analysis on the single‐gene level was performed to identify genes differentially expressed in ultralow risk tumors compared to all other ER‐positive tumors of low or high risk. Overall, 706 genes were significantly (FDR < 0.001) differentially expressed in ultralow risk tumors as compared to all other ER‐positive tumors of low or high risk (Table S4). Of these, 454 genes were expressed at lower levels in ultralow risk tumors and 252 genes at higher expression levels.

**FIGURE 3:**
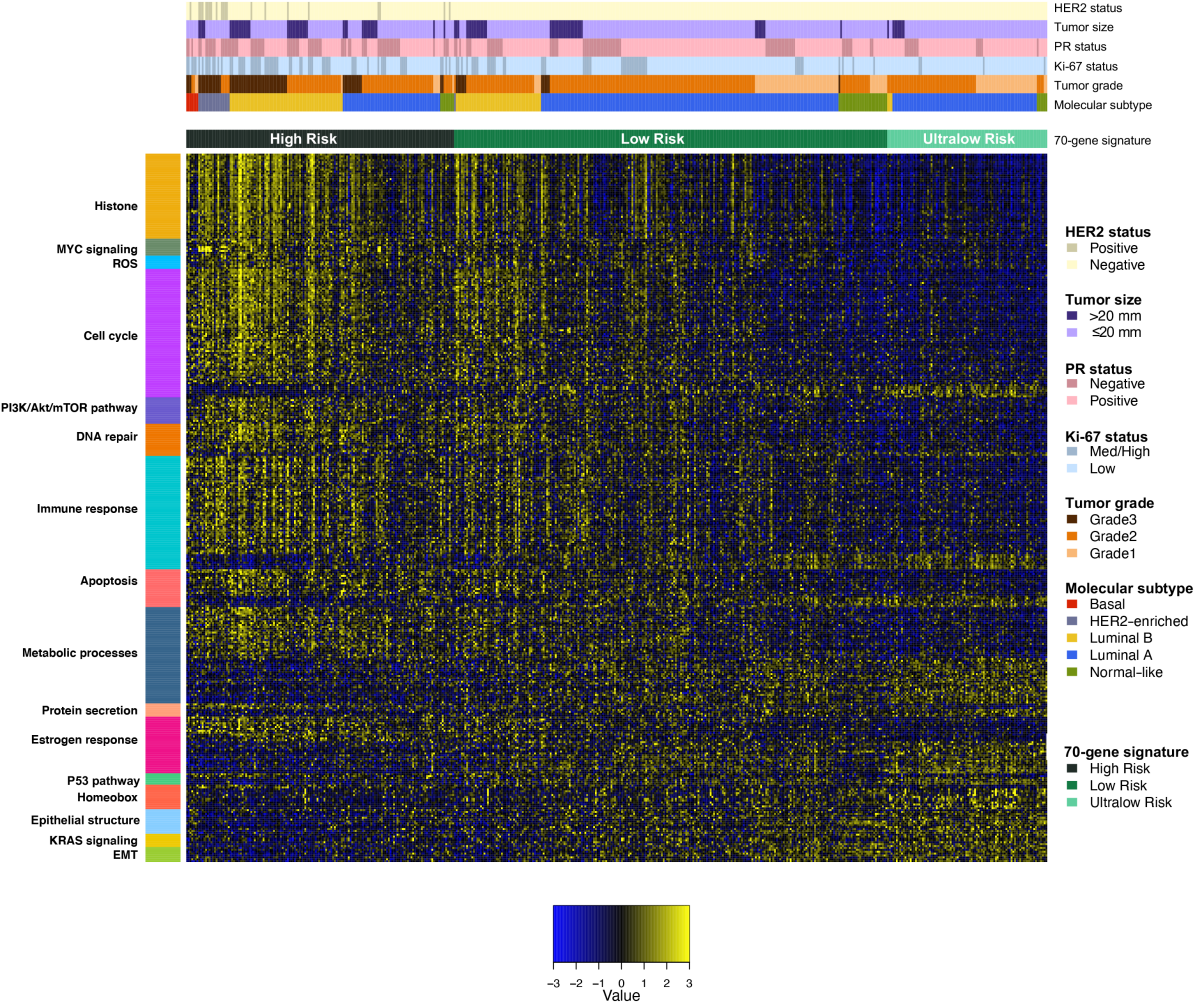
Differentially expressed genes in ultralow risk breast cancer tumors. Heatmap of genes differentially expressed between ultralow risk tumors and all other ER‐positive tumors (of low or high risk). Patients (columns) are ordered by the 70‐gene signature classification, molecular subtype, tumor grade, Ki‐67 status, PR status, tumor size and HER2 status. Genes are categorized by their involvement in different biological processes or pathways (Hallmark gene sets), and ordered by *t*‐statistics between and within each gene category. EMT, epithelial‐to‐mesenchymal transition; ROS, reactive oxygen species

To further understand the biological function associated with ultralow risk definition, the 706 differentially expressed genes were categorized into different cancer‐related Hallmark gene sets (Table S4). Figure 3 shows a heatmap of a subset of the differentially expressed genes ordered by their Hallmark gene category and with patients ordered by clinical and molecular characteristics. The gene expression patterns in the heatmap were clearly different between the ultralow risk and high risk tumors, whereas the tumors classified as low risk showed a more heterogenous gene expression pattern (Figure 3). Consistent with the previous results of the multigene modules, ultralow risk tumors showed lower expression levels of genes associated with the PI3K/Akt/mTOR pathway and immune response (Figure 3 and Table S4). Moreover, ultralow risk tumors generally expressed lower levels of genes coding for histones, MYC‐signaling, reactive oxygen species, cell cycle, DNA repair and apoptosis (Figure 3 and Table S4). Genes coding for homeobox proteins, epithelial structure, KRAS‐signaling and epithelial‐to‐mesenchymal transition (EMT) were expressed in higher levels in ultralow risk tumors. Genes involved in different metabolic processes, protein secretion, estrogen response or P53 pathway showed both higher and lower expression levels in ultralow risk tumors.

Further gene set enrichment analysis (GSEA) showed significant (FDR < 0.05) enrichment of gene sets consistent with previous analyses (Table S5). The gene sets MYC‐signaling, cell cycle, DNA‐repair, unfolded protein response, PI3K/Akt/mTOR‐pathway, immune response and apoptosis were downregulated in ultralow risk tumors, and EMT upregulated. Furthermore, the GSEA also showed the gene sets metabolic processes and P53‐pathway to be downregulated in ultralow risk tumors, whereas myogenesis, hedgehog signaling and estrogen response were upregulated.

## 4 DISCUSSION

We have previously shown that the ultralow risk threshold of the 70‐gene signature identifies patients at minimal long‐term risk of death from breast cancer.^9^ To further understand the long‐term risk of breast cancer, we here aimed to explore the clinical and molecular characteristics of primary ultralow risk tumors from the STO‐3 trial. Our study shows that ultralow risk tumors are significantly more likely to be of a smaller tumor size, of lower tumor grade, PR‐positive, HER2‐negative and Ki‐67‐low, compared to other ER‐positive tumors. Moreover, ultralow risk tumors exhibited substantially different expressions of “hallmark” gene sets as well as other important cancer‐related biological processes or pathways.

The risk of distant recurrences in ER‐positive breast cancer remains steady decades after primary diagnosis.^1^, ^2^, ^3^, ^4^, ^5^, ^6^ Consequently, it is important to identify distinct biological characteristics that predict patients' long‐term recurrence risk to improve our understanding and distinguish our management of nonfatal vs fatal breast cancers—something that has proven a great challenge. Here, our findings suggest that ultralow risk tumors have differential tumor characteristics as compared to other ER‐positive tumors, including luminal A molecular subtype tumors which are generally considered to be of low risk. Noteworthy, far from all luminal A molecular subtype tumors (1 out of 4) are classified as ultralow risk, which indicates that the ultralow risk classification differs from the molecular subtyping. We have previously shown that patients with luminal A tumors have a long‐term risk of distant recurrences, which is reduced by tamoxifen therapy.^3^ Thus, the luminal A molecular subtype may yet consist of a heterogeneous group of tumors, were the ultralow risk threshold can be used to identify patients with a very low long‐term risk. Therefore, the 70‐gene signature ultralow risk threshold may be helpful to minimize overtreatment and reassure patients in terms of their low long‐term risk of distant recurrences, together with more traditionally used clinical markers,^1^ and other prognostic predictors such as urokinase plasminogen activator (uPA) levels and its inhibitor,^35^ molecular subtype classification and risk of recurrence (ROR) score^36^ and ER‐intratumor heterogeneity levels.^2^

Ultralow risk tumors differ significantly from other ER‐positive tumors in relation to the clinically used breast cancer markers. PR and Ki‐67 have prognostic value in breast cancer,^5^, ^37^, ^38^ and we show that ultralow risk tumors are significantly more likely to be PR‐positive and Ki‐67‐low (<15%), as determined by IHC. Together with lower expression of genes involved in the cell cycle and the proliferation‐associated multigene module AURKA, this suggests that lower proliferation is an important clinical characteristic of ultralow risk tumors. Interestingly, the expression scores of the multigene module reflecting HER2/*ERBB2*‐signaling significantly differ between the ultralow risk tumors as compared to the other ER‐positive tumor groups. However, since this difference was not observed by the HER2 IHC assay (with no ultralow risk tumors categorized as HER2‐positive, whereas 16% were categorized as high HER2‐expression by the HER2/*ERBB2* multigene module) this finding should be confirmed in further studies. Furthermore, ultralow risk tumors exhibited higher expression scores of the *ESR1*‐module, indicating higher ER expression which is associated with better prognosis.^27^

Ultralow risk tumors differ significantly from other ER‐positive tumors on the molecular level by the expression of cancer‐associated biological processes and pathways. In brief, genes involved in the PI3K/Akt/mTOR pathway, which has a role in endocrine sensitivity and breast cancer survival,^39^, ^40^ show lower expression in ultralow risk tumors, as observed both by the multigene module AKT/mTOR and on the single‐gene level. Furthermore, loss of PTEN, a PI3K‐regulator and tumor suppressor, is more frequent in the ultralow risk tumors, and PIK3CA‐mutations, which have been associated with more favorable outcome in ER‐positive breast cancer,^41^ are more common. Moreover, the difference in expression in transcriptionally important histone genes and homeobox genes implicate a potentially altered transcriptional and epigenetic state in ultralow risk tumors.^42^

Lower expression of immune response genes is recognized in ultralow risk tumors, as observed by the multigene modules IMMUNE1 and IMMUNE2 and on the single‐gene level. While the immune response mostly has been investigated in the context of ER‐negative or HER2‐positive breast cancer were higher expression often has been associated with better survival and reduced metastatic risk,^28^, ^43^ it has also been associated with better pathologic complete response (pCR) in ER‐positive/HER2‐negative patients.^29^ Contrary to this, our observed lower expression of immune response genes in ultralow risk tumors is a new and clinically provocative finding that deserves further validation.

Another finding in our study indicate that ultralow risk tumors exhibit lower expression scores of the IGF1‐pathway‐associated module, which is involved in breast cancer development and progression.^44^, ^45^ Interestingly yet relatively small gene groups identified as differentially expressed in ultralow risk tumors include MYC, reactive oxygen species, P53, KRAS and EMT. These, as well as genes involved in metabolic processes and estrogen response, which showed both lower and higher expression levels in ultralow risk tumors, require further analyses to understand their role in relation to long‐term metastatic risk. Noteworthy, even though higher expression of EMT genes might be a sign of metastatic development,^46^ a majority of the differentially expressed genes in ultralow risk tumors associated with EMT have been recognized as tumor‐suppressor genes, including *TPM1*, *WNT5A*, *TGFBR3*, *SLIT3* and *PDLIM4*.^47^, ^48^, ^49^, ^50^, ^51^

To summarize, our study suggests an ultralow risk phenotype characterized by clinically used breast cancer markers (tumor size, tumor grade, PR status, HER2 status and Ki‐67 status) and molecular characteristics. Importantly, the ultralow risk classification differs from the luminal A molecular subtyping. Furthermore, ultralow risk tumors show lower activation of the PI3K/Akt/mTOR‐pathway, immune response and IGF1‐pathway and lower expression levels of genes involved in proliferation, cell cycle, DNA repair and apoptosis and higher expression levels of EMT tumor suppressor genes. Also, an altered transcriptional state in ultralow risk tumors is suggested, including lower expression levels of genes coding for histones and higher expression levels of homeobox genes, which less is known about. These findings are supported by analysis of multigene expression modules and the identification of differentially expressed genes and their associated Hallmark gene sets.

Our study was conducted using tumors from postmenopausal patients with generally low metastatic risks given their lymph‐node negative status and smaller (≤30 mm) tumors. There are limitations to our study. The STO‐3 trial was conducted mainly prior to the introduction of mammographic screening in Sweden; therefore, the majority of the breast tumors were clinically detected. Modern mammographic screening has increased the number of newly diagnosed low‐risk breast tumors, thus, a better clinical understanding of low‐risk tumors characteristics is needed. As with most long‐term follow‐up studies, clinical recommendations for disease management and treatment have changed since the trial. Molecular analysis was possible for approximately half of the tumors in STO‐3; however, we have confirmed that patient and tumor characteristics were well balanced to the original STO‐3 cohort.^23^ Further, when dealing with IHC assays, there is often some degree of subjective inaccuracy. However, the clinical IHC markers were recently re‐assessed at a single medical laboratory by dedicated breast cancer pathologists.^52^ Despite our relatively small study size with 98 ultralow risk tumors, we were able to identify significant and informative differences between the analyzed groups. Another clear strength of our study includes the recent performance and annotation of genome‐wide gene expression analyses.

## 5 CONCLUSIONS

Ultralow risk tumors, associated with minimal long‐term risk of fatal breast cancer, have distinct clinical, biological and molecular characteristics as compared to other ER‐positive tumors, including luminal A molecular subtype tumors that generally are considered as a low‐risk disease. A better understanding of the characteristics of breast cancer tumors of very low long‐term risk of metastatic disease is important to improve our prediction of fatal vs nonfatal disease.

## CONFLICT OF INTEREST

Dr van 't Veer is a cofounder, stockholder and part‐time employee of Agendia. All remaining authors declare no potential conflicts of interest.

## AUTHOR CONTRIBUTIONS

Conceptualization: Annelie Johansson, Nancy Yiu‐Lin Yu, Adina Iftimi, Laura van 't Veer, Christopher C. Benz, Laura J. Esserman, Christina Yau and Linda S. Lindström. Data curation: Bo Nordenskjöld, Tommy Fornander, Olle Stål, Laura J. Esserman and Linda S. Lindström. Formal analysis: Annelie Johansson, Nancy Yiu‐Lin Yu, Adina Iftimi, Christina Yau and Linda S. Lindström. Funding acquisition: Tommy Fornander, Olle Stål, Laura J. Esserman and Linda S. Lindström. Investigation: Annelie Johansson, Nancy Yiu‐Lin Yu, Adina Iftimi, Gizeh Perez‐Tenorio, Christina Yau and Linda S. Lindström. Methodology: Annelie Johansson, Nancy Yiu‐Lin Yu, Adina Iftimi, Christina Yau and Linda S. Lindström. Project administration: Annelie Johansson and Linda S. Lindström. Resources: Bo Nordenskjöld, Tommy Fornander, Olle Stål and Linda S. Lindström. Software: Annelie Johansson, Nancy Yiu‐Lin Yu, Adina Iftimi, Nicholas P. Tobin, Christina Yau and Linda S. Lindström. Supervision: Annelie Johansson and Linda S. Lindström. Visualization: Annelie Johansson, Nancy Yiu‐Lin Yu, Adina Iftimi, Gizeh Perez‐Tenorio, Christina Yau and Linda S. Lindström. Writing ‐ original draft: Annelie Johansson, Nancy Yiu‐Lin Yu and Linda S. Lindström. Writing ‐ review & editing: All authors.

## ETHICS STATEMENT

The STO‐3 trial was approved by the ethics committee at Karolinska Institutet and participants provided oral consent. The trial was conducted at the Regional Cancer Center Stockholm‐Gotland, Sweden, and began in 1976, which was well before trial registration started in Sweden; therefore, information on trial registration number is not available.

## Supporting information


**Appendix** S1: Supporting Information

## Data Availability

The raw RNA microarray data generated in our study is deposited on a secure Swedish server and has been assigned a DOI (https://doi.org/10.5878/3vxa-3c28). Data access requests may be submitted to the Swedish National Data Service through the DOI link, and will be reviewed by the STO Trialists Group. The R‐code to reproduce the results and figures are publicly available at https://github.com/annelieewa/Ultralow_risk_STO3. Other data that support the findings of our study are available from the corresponding author upon request and with the permission of the STO Trialists Group.
