# Supplementary material for: Clinical and molecular characteristics of estrogen receptor‐positive ultralow risk breast cancer tumors identified by the 70‐gene signature
Source: Int J Cancer. 2022 Mar 7;150(12):2072–82. doi: 10.1002/ijc.33969 (PMC9083187; doi:10.1002/ijc.33969)
Supplement: Supplementary file 1 — Appendix S1: Supporting Information [file IJC-150-2072-s001.pdf]

SUPPLEMENTARY MATERIAL

Clinical and Molecular Characteristics of ER-Positive Ultralow Risk Breast Cancer Tumors Identified by the 70-Gene Signature

Annelie Johansson,* Nancy Yiu-Lin Yu, Adina Iftimi, Nicholas P Tobin, Laura van 't Veer, Bo Nordenskjöld, Christopher C Benz, Tommy Fornander, Gizeh Perez-Tenorio, Olle Stål, Laura J Esserman, Christina Yau, and Linda S Lindström

* Correspondence: annelie.johansson@ki.se

Table of contents

Supplementary Materials & Methods

Supplementary Table S1. Gene groups for the heatmap

Supplementary Table S2. Molecular subtypes of ultralow risk breast cancer tumors

Supplementary Table S3. Multi-gene module expression scores in ultralow risk breast cancer tumors

Supplementary Table S4. Differentially expressed genes in ultralow risk breast cancer tumors

Supplementary Table S5. Result from Gene Set Enrichment Analysis (GSEA)

SUPPLEMENTARY MATERIALS & METHODS

Randomization in the STO-3 trial

Randomization was by telephone to a central office and done using balanced lists with a random number table (permuted block technique). The allocated treatment was revealed to the clinician after randomization was registered at central office.[1]

Molecular subtypes

Breast cancer tumors in STO-3 were classified according to the molecular PAM50 subtype classification.[2] Gene expressions from each chip were normalized using log2-scaled upper quartile normalization. A patient subset was generated using all 113 ER-negative patient samples in STO-3, with 113 ER-positive patient samples randomly selected to mirror the ER distribution in the PAM50 classifier training set. Gene expression values of all patient samples (n=226) were adjusted to the median gene values. Microarray probes from the patient samples were mapped to the PAM50 classifier by Human Genome Organization Gene Nomenclature (HGNC) symbols. Expression values from genes represented by multiple probes were computed by averaging expressions from the probes, as per recommended for long oligo platforms.

Multi-gene expression modules

To investigate the tumor biology of the breast cancer samples, 19 multi-gene expression modules that serve as proxies of biological processes and pathway activation were assessed.[3-5] Desmedt *et al.* generated 7 multi-gene modules (AURKA, CASP3, ERBB2, ESR1, IMMUNE2 [also known as STAT1], STROMA2 [also known as PLAU], VEGF) that serve as proxies for proliferation, apoptosis, ERBB2/HER2 signaling, ER signaling, immune response, tumor invasion/metastasis, and angiogenesis, respectively.[3] Ignatiadis *et al.* further evaluated 10 pathway-related gene signatures.[4] These included activation status of the oncogenic pathways RAS, SRC, MYC, E2F3, and Wnt/beta-catenin (BETAC), which were originally generated from Bild *et al.,*[6] as well as gene signatures serving as proxies for Akt/mTOR pathway activation,[7] IGF1 pathway,[8] MAPK pathway,[9] mutation of PIK3CA,[10] and oncogenic pathway activation due to PTEN loss.[11] Further, multi-gene modules for stromal environment (STROMA1[12]) and immune cells infiltration (IMMUNE1[13]) were included. Gene modules

in which the low scores were suggested to be associated with clinical aggressiveness and worse prognosis included CASP3, ESR1, PIK3CA, and VEGF; for all others, high scores were associated with clinical aggressiveness.

Annotation of genes differentially expressed in ultralow risk tumors

Differentially expressed genes were categorized by using the MsigDB Hallmark gene sets (v6.2),[14] as well as the gene sets from the Gene Ontology (GO) biological processes (C5 collection v6.2).[15] The Hallmark collection contains 50 gene sets, which we grouped into 27 larger gene sets (see **Supplementary Table S1**), of which the largest groups being cell cycle (containing the hallmarks E2F targets, G2M checkpoint, mitotic spindle), immune response (containing the hallmarks allograft rejection, complement, IL2/STAT5 signaling, IL6/JAK/STAT3 signaling, inflammatory response, IFN-α [interferon alpha] response, IFN-γ [interferon gamma] response, TGF-β [transforming growth factor beta] signaling, and TNF-α [tumor necrosis factor alpha] signaling via NFκB), and metabolic processes (containing the hallmarks bile acid metabolism, cholesterol homeostasis, fatty acid metabolism, heme metabolism, oxidative phosphorylation, peroxisome, and xenobiotic metabolism). Some other gene sets were combined in pairs (e.g. early and late estrogen response), see **Supplementary Table S1**. Furthermore, genes coding for histones or homeoboxes were identified by their gene names. Lastly, similar or identical biological processes in the GO gene sets were identified for each of the 27 hallmark groups, e.g. GO_ANGIOGENESIS for HALLMARK_ANGIOGENESIS, see **Supplementary Table S1**.

Genes were categorized as following: 1) if the gene was coding for histone or homeobox, it was categorized as this. 2) If the gene belonged to one hallmark gene set, this one was used. 3) If a gene belonged to one GO gene set, and no hallmark gene set, the GO gene set was used. 4) If any gene set was overlapping between the hallmark or GO, that gene set was used. 5) After this, small gene sets with less than 3 genes in the gene set were identified and step 2-3 were repeated without the small groups. 6) Lastly, if the gene was involved in estrogen response, we categorized it as this.

Heatmap code was modified from: https://raw.githubusercontent.com/obigriffith/biostar-tutorials/master/Heatmaps/heatmap.3.R. Genes not belonging to any of the main cancer-related categories were not shown in the heatmap, or groups with fewer than 5 genes in the group.

Supplementary Table S1. Gene groups for the heatmap. List of gene groups, Hallmark gene sets, and Gene Ontology biological processes used to categorize the differentially expressed genes for the heatmap.

**Gene Category**	**Hallmark**	**Gene Ontology Biological Processes**
ADIPOGENESIS	HALLMARK_ADIPOGENESIS	GO_ADIPOSE_TISSUE_DEVELOPMENT
ANDROGEN_RESPONSE	HALLMARK_ANDROGEN_RESPONSE	GO_ANDROGEN_RECEPTOR_SIGNALING_PATHWAY; GO_REGULATION_OF_ANDROGEN_RECEPTOR_SIGNALING_PATHWAY
ANGIOGENESIS	HALLMARK_ANGIOGENESIS	GO_ANGIOGENESIS
APOPTOSIS	HALLMARK_APOPTOSIS	GO_CELL_DEATH; GO_REGULATION_OF_CELL_DEATH
CELL_CYCLE	HALLMARK_E2F_TARGETS	GO_CELL_CYCLE_G1_S_PHASE_TRANSITION; GO_REGULATION_OF_CELL_CYCLE_G1_S_PHASE_TRANSITION
CELL_CYCLE	HALLMARK_G2M_CHECKPOINT	GO_CELL_CYCLE_G2_M_PHASE_TRANSITION; GO_REGULATION_OF_CELL_CYCLE_G2_M_PHASE_TRANSITION
CELL_CYCLE	HALLMARK_MITOTIC_SPINDLE	GO_MITOTIC_SPINDLE_ASSEMBLY; GO_MITOTIC_SPINDLE_ORGANIZATION
COAGULATION	HALLMARK_COAGULATION	GO_REGULATION_OF_COAGULATION
DNA_REPAIR	HALLMARK_DNA_REPAIR	GO_DNA_REPAIR; GO_REGULATION_OF_DNA_REPAIR
EPITHELIAL_MESENCHYMAL_TRANSITION	HALLMARK_EPITHELIAL_MESENCHYMAL_TRANSITION	GO_EPITHELIAL_TO_MESENCHYMAL_TRANSITION; GO_REGULATION_OF_EPITHELIAL_TO_MESENCHYMAL_TRANSITION
EPITHELIAL_STRUCTURE	HALLMARK_APICAL_JUNCTION	GO_CELL_JUNCTION_ASSEMBLY; GO_REGULATION_OF_CELL_JUNCTION_ASSEMBLY
EPITHELIAL_STRUCTURE	HALLMARK_APICAL_SURFACE	GO_ESTABLISHMENT_OR_MAINTENANCE_OF_CELL_POLARITY; GO_REGULATION_OF_ESTABLISHMENT_OR_MAINTENANCE_OF_CELL_POLARITY
ESTROGEN_RESPONSE	HALLMARK_ESTROGEN_RESPONSE_EARLY; ESTROGEN_RESPONSE_LATE	GO_RESPONSE_TO_ESTROGEN; GO_INTRACELLULAR_ESTROGEN_RECEPTOR_SIGNALING_PATHWAY; GO_REGULATION_OF_INTRACELLULAR_ESTROGEN_RECEPTOR_SIGNALING_PATHWAY
HEDGEHOG_SIGNALING	HALLMARK_HEDGEHOG_SIGNALING	NONE
HYPOXIA	HALLMARK_HYPOXIA	GO_REGULATION_OF_CELLULAR_RESPONSE_TO_HYPOXIA
IMMUNE	HALLMARK_ALLOGRAFT_REJECTION	NONE
IMMUNE	HALLMARK_IL2_STAT5_SIGNALING	GO_REGULATION_OF_TYROSINE_PHOSPHORYLATION_OF_STAT5_PROTEIN
IMMUNE	HALLMARK_IL6_JAK_STAT3_SIGNALING	GO_RESPONSE_TO_INTERLEUKIN_6; GO_REGULATION_OF_TYROSINE_PHOSPHORYLATION_OF_STAT3_PROTEIN
IMMUNE	HALLMARK_INFLAMMATORY_RESPONSE	GO_INFLAMMATORY_RESPONSE; GO_REGULATION_OF_INFLAMMATORY_RESPONSE
IMMUNE	HALLMARK_INTERFERON_ALPHA_RESPONSE	GO_RESPONSE_TO_INTERFERON_ALPHA
IMMUNE	HALLMARK_INTERFERON_GAMMA_RESPONSE	GO_RESPONSE_TO_INTERFERON_GAMMA; GO_REGULATION_OF_RESPONSE_TO_INTERFERON_GAMMA

IMMUNE	HALLMARK_TGF_BETA_SIGNALING	GO_RESPONSE_TO_TRANSFORMING_GROWTH_FACTOR_BETA; GO_REGULATION_OF_CELLULAR_RESPONSE_TO_TRANSFORMING_GROWTH_FACTOR_BETA_STIMULUS
IMMUNE	HALLMARK_TNFA_SIGNALING_VIA_NFKB	GO_TUMOR_NECROSIS_FACTOR_MEDIATED_SIGNALING_PATHWAY; GO_REGULATION_OF_TUMOR_NECROSIS_FACTOR_MEDIATED_SIGNALING_PATHWAY
KRAS	KRAS_SIGNALING_DN: KRAS_SIGNALING_UP	NONE
METABOLIC	HALLMARK_BILE_ACID_METABOLISM	GO_BILE_ACID_METABOLIC_PROCESS
METABOLIC	HALLMARK_CHOLESTEROL_HOMEOSTASIS	GO_REGULATION_OF_CHOLESTEROL_HOMEOSTASIS
METABOLIC	HALLMARK_COMPLEMENT	GO_COMPLEMENT_ACTIVATION
METABOLIC	HALLMARK_FATTY_ACID_METABOLISM	GO_FATTY_ACID_METABOLIC_PROCESS; GO_REGULATION_OF_FATTY_ACID_METABOLIC_PROCESS
METABOLIC	HALLMARK_GLYCOLYSIS	GO_GLUCOSE_METABOLIC_PROCESS; GO_REGULATION_OF_GLUCOSE_METABOLIC_PROCESS; GO_REGULATION_OF_GLUCONEOGENESIS
METABOLIC	HALLMARK_HEME_METABOLISM	GO_HEME_METABOLIC_PROCESS
METABOLIC	HALLMARK_OXIDATIVE_PHOSPHORYLATION	GO_OXIDATIVE_PHOSPHORYLATION; GO_REGULATION_OF_OXIDATIVE_PHOSPHORYLATION
METABOLIC	HALLMARK_PEROXISOME	GO_PEROXISOME_ORGANIZATION
METABOLIC	HALLMARK_XENOBIOTIC_METABOLISM	GO_RESPONSE_TO_XENOBIOTIC_STIMULUS
MYC_TARGETS	MYC_TARGETS_V1: MYC_TARGETS_V2	NONE
MYOGENESIS	HALLMARK_MYOGENESIS	GO_REGULATION_OF_SKELETAL_MUSCLE_TISSUE_DEVELOPMENT
NOTCH_SIGNALING	HALLMARK_NOTCH_SIGNALING	GO_NOTCH_SIGNALING_PATHWAY; GO_REGULATION_OF_NOTCH_SIGNALING_PATHWAY
P53_PATHWAY	HALLMARK_P53_PATHWAY	GO_SIGNAL_TRANSDUCTION_BY_P53_CLASS_MEDIATOR; GO_REGULATION_OF_SIGNAL_TRANSDUCTION_BY_P53_CLASS_MEDIATOR
PANCREAS_BETA_CELLS	HALLMARK_PANCREAS_BETA_CELLS	GO_INSULIN_SECRETION; GO_POSITIVE_REGULATION_OF_INSULIN_SECRETION
PI3K_AKT_MTOR_SIGNALING	HALLMARK_MTORC1_SIGNALING	NONE
PI3K_AKT_MTOR_SIGNALING	HALLMARK_PI3K_AKT_MTOR_SIGNALING	GO_PHOSPHATIDYLINOSITOL_3_KINASE_SIGNALING; GO_REGULATION_OF_PHOSPHATIDYLINOSITOL_3_KINASE_SIGNALING; GO_PROTEIN_KINASE_B_SIGNALING GO_REGULATION_OF_PROTEIN_KINASE_B_SIGNALING; GO_TOR_SIGNALING; GO_REGULATION_OF_TOR_SIGNALING
PROTEIN_SECRETION	HALLMARK_PROTEIN_SECRETION	GO_PROTEIN_SECRETION; GO_REGULATION_OF_PROTEIN_SECRETION
REACTIVE_OXYGEN_SPECIES_PATHWAY	HALLMARK_REACTIVE_OXYGEN_SPECIES_PATHWAY	GO_RESPONSE_TO_REACTIVE_OXYGEN_SPECIES; GO_REGULATION_OF_RESPONSE_TO_REACTIVE_OXYGEN_SPECIES
SPERMATOGENESIS	HALLMARK_SPERMATOGENESIS	NONE

UNFOLDED_PROTEIN_RESPONSE	HALLMARK_UNFOLDED_PROTEIN_RESPONSE	GO_REGULATION_OF_ENDOPLASMIC_RETICULUM_UNFOLDED_PROTEIN_RESPONSE
UV_RESPONSE	HALLMARK_UV_RESPONSE_DN: HALLMARK_UV_RESPONSE_UP	GO_RESPONSE_TO_UV
WNT_BETA_CATENIN_SIGNALING	HALLMARK_WNT_BETA_CATENIN_SIGNALING	GO_WNT_SIGNALING_PATHWAY; GO_REGULATION_OF_WNT_SIGNALING_PATHWAY

Supplementary Table S2. Molecular subtypes of ultralow risk breast cancer tumors.

Molecular Subtype [a]		ER-positive tumors	
		Ultralow risk (n=98)	Low or high risk (n=440)
**Luminal A (n=336)**	Yes	87	249
	No	11	191
**Luminal B (n=126)**	Yes	4	122
	No	94	318
**Normal-like (n=48)**	Yes	7	41
	No	91	399
**HER2-enriched (n=21)**	Yes	0	21
	No	98	419
**Basal (n=7)**	Yes	0	7
	No	98	433

[a] Molecular subtyping by the PAM50 classification

Supplementary Table S3. Multi-gene module expression scores in ultralow risk breast cancer tumors. The expression scores of multi-gene modules in ultralow risk breast cancer tumors versus all other ER-positive tumors (of low or high risk), and ER-positive luminal A and luminal B molecular subtype tumors (of low or high risk) by Fisher's exact test. Significant P-values (*P*<0.05) are marked in bold.

Multi-Gene Module Expression Scores		Ultralow risk tumors (n=98)	ER-positive tumors of low or high risk					
			All other ER-positive tumors (n=440)		Luminal A tumors (n=249)		Luminal B tumors (n=122)	
		No (%)	No (%)	P	No (%)	P	No (%)	P
**AKT-MTOR (pathway)**								
	low	81 (82.7)	302 (68.6)	**0.006**	175 (70.3)	**0.021**	70 (57.4)	**<0.001**
	high	17 (17.3)	138 (31.4)		74 (29.7)		52 (42.6)	
**AURKA (proliferation)**								
	low	96 (98.0)	303 (68.9)	**<0.001**	207 (83.1)	**<0.001**	43 (35.2)	**<0.001**
	high	2 (2.0)	137 (31.1)		42 (16.9)		79 (64.8)	
**BETAC (pathway)**								
	low	71 (72.4)	298 (67.7)	0.401	185 (74.3)	0.786	76 (62.3)	0.116
	high	27 (27.6)	142 (32.3)		64 (25.7)		46 (37.7)	
**CASP3 (apoptosis)**								
	high	56 (57.1)	293 (66.6)	0.080	155 (62.2)	0.395	93 (76.2)	**0.004**
	low	42 (42.9)	147 (33.4)		94 (37.8)		29 (23.8)	
**E2F3 (pathway)**								
	low	77 (78.6)	282 (64.1)	**0.006**	171 (68.7)	0.086	66 (54.1)	**<0.001**
	high	21 (21.4)	158 (35.9)		78 (31.3)		56 (45.9)	
**ERBB2 (HER2-signalling)**								
	low	82 (83.7)	285 (64.8)	**<0.001**	173 (69.5)	**0.007**	72 (59.0)	**<0.001**
	high	16 (16.3)	155 (35.2)		76 (30.5)		50 (41.0)	
**ESR1 (ER-signalling)**								
	high	91 (92.9)	340 (77.3)	**<0.001**	217 (87.1)	0.185	106 (86.9)	0.186
	low	7 (7.1)	100 (22.7)		32 (12.9)		16 (13.1)	
**IGF1 (pathway)**								
	low	96 (98.0)	320 (72.7)	**<0.001**	215 (86.3)	**0.001**	63 (51.6)	**<0.001**
	high	2 (2.0)	120 (27.3)		34 (13.7)		59 (48.4)	
**IMMUNE1 (immune response)**								
	low	80 (81.6)	291 (66.1)	**0.002**	171 (68.7)	**0.016**	80 (65.6)	**0.009**
	high	18 (18.4)	149 (33.9)		78 (31.3)		42 (34.4)	
**IMMUNE2 (immune response)**								
	low	92 (93.9)	302 (68.6)	**<0.001**	192 (77.1)	**<0.001**	70 (57.4)	**<0.001**
	high	6 (6.1)	138 (31.4)		57 (22.9)		52 (42.6)	
**MAPK (pathway)**								
	low	73 (74.5)	339 (77.0)	0.599	197 (79.1)	0.390	110 (90.2)	**0.003**
	high	25 (25.5)	101 (23.0)		52 (20.9)		12 (9.8)	

**MYC (pathway)**							
low	80 (81.6)	300 (68.2)	**0.010**	190 (76.3)	0.317	58 (47.5)	**<0.001**
high	18 (18.4)	140 (31.8)		59 (23.7)		64 (52.5)	
**PIK3CA-mutations**							
high	90 (91.8)	300 (68.2)	**<0.001**	190 (76.3)	**0.001**	67 (54.9)	**<0.001**
low	8 (8.2)	140 (31.8)		59 (23.7)		55 (45.1)	
**PTEN-loss**							
low	97 (99.0)	294 (66.8)	**<0.001**	205 (82.3)	**<0.001**	39 (32.0)	**<0.001**
high	1 (1.0)	146 (33.2)		44 (17.7)		83 (68.0)	
**RAS (pathway)**							
low	71 (72.4)	316 (71.8)	1.000	178 (71.5)	0.895	93 (76.2)	0.537
high	27 (27.6)	124 (28.2)		71 (28.5)		29 (23.8)	
**SRC (pathway)**							
low	73 (74.5)	302 (68.6)	0.276	191 (76.7)	0.676	78 (63.9)	0.109
high	25 (25.5)	138 (31.4)		58 (23.3)		44 (36.1)	
**STROMA1 (stromal invasion)**							
low	55 (56.1)	289 (65.7)	0.082	146 (58.6)	0.718	94 (77.0)	**0.001**
high	43 (43.9)	151 (34.3)		103 (41.4)		28 (23.0)	
**STROMA2 (stromal invasion)**							
low	57 (58.2)	298 (67.7)	0.077	160 (64.3)	0.325	94 (77.0)	**0.003**
high	41 (41.8)	142 (32.3)		89 (35.7)		28 (23.0)	
**VEGF (angiogenesis)**							
high	66 (67.3)	276 (62.7)	0.418	167 (67.1)	1.000	80 (65.6)	0.886
low	32 (32.7)	164 (37.3)		82 (32.9)		42 (34.4)	

Supplementary Table S4. Differentially expressed genes in ultralow risk breast cancer tumors. List of genes differentially expressed between ultralow risk tumors and all other ER-positive tumors (of low or high risk).

ProbeID	Gene	t-statistics	Ultralow expression	*P*-value	FDR	Sensitivity	Hallmark Gene Category
A_24_P172990	AARS	-4.58	Lower expression	<1.00E-07	<1.00E-07	0.041	IMMUNE
A_23_P8754	AASS	4.29	Higher expression	3.94E-06	9.39E-05	0.056	
A_23_P362712	ABHD11	-4.73	Lower expression	<1.00E-07	<1.00E-07	0.036	
A_23_P69339	ACAA1	4.59	Higher expression	<1.00E-07	<1.00E-07	0.041	METABOLIC
A_23_P207650	ACADVL	5.14	Higher expression	<1.00E-07	<1.00E-07	0.022	METABOLIC
A_33_P3319155	ACP1	-4.2	Lower expression	9.85E-06	1.86E-04	0.062	
A_23_P56798	ACP1	-4.15	Lower expression	1.38E-05	2.52E-04	0.064	
A_23_P69249	ACTL6A	-3.94	Lower expression	4.93E-05	7.43E-04	0.078	DNA_REPAIR
A_23_P502343	ADAM33	4.02	Higher expression	3.35E-05	5.54E-04	0.072	
A_24_P134319	ADNP	-4.62	Lower expression	<1.00E-07	<1.00E-07	0.039	APOPTOSIS
A_24_P33770	AFAP1L2	4.16	Higher expression	9.85E-06	1.86E-04	0.063	EPITHELIAL_STRUCTURE
A_24_P57367	AHCY	-4.96	Lower expression	<1.00E-07	<1.00E-07	0.027	IMMUNE
A_33_P3307253	AK5	5.06	Higher expression	<1.00E-07	<1.00E-07	0.024	
A_24_P342632	AK5	3.98	Higher expression	4.14E-05	6.47E-04	0.076	
A_33_P3379644	ALDH1A1	4.6	Higher expression	<1.00E-07	<1.00E-07	0.04	METABOLIC
A_23_P152984	ALYREF	-4.88	Lower expression	<1.00E-07	<1.00E-07	0.03	
A_23_P14083	AMIGO2	-4.04	Lower expression	3.35E-05	5.54E-04	0.072	EPITHELIAL_STRUCTURE
A_23_P12533	ANKRD30A	6.36	Higher expression	<1.00E-07	<1.00E-07	0.008	
A_33_P3368985	ANKRD30A	5.44	Higher expression	<1.00E-07	<1.00E-07	0.018	
A_33_P3277659	ANKRD30B	6.45	Higher expression	<1.00E-07	<1.00E-07	0.008	
A_33_P3343452	ANKRD30B	4.32	Higher expression	3.94E-06	9.39E-05	0.054	
A_24_P221007	ANKRD30B	4.15	Higher expression	1.38E-05	2.52E-04	0.064	
A_33_P3343457	ANKRD30B	3.9	Higher expression	5.52E-05	7.98E-04	0.082	
A_24_P281665	ANKRD30BL	4.57	Higher expression	<1.00E-07	<1.00E-07	0.041	
A_23_P321496	ANKRD30BP2	4.78	Higher expression	<1.00E-07	<1.00E-07	0.033	
A_32_P192765	ANKRD30BP3	5.25	Higher expression	<1.00E-07	<1.00E-07	0.02	
A_23_P157099	ANLN	-5.92	Lower expression	<1.00E-07	<1.00E-07	0.012	CELL_CYCLE
A_23_P356684	ANLN	-4.2	Lower expression	9.85E-06	1.86E-04	0.061	CELL_CYCLE
A_24_P109214	APOC1	-4.52	Lower expression	3.94E-06	9.39E-05	0.043	IMMUNE
A_33_P3223592	APOE	-4.99	Lower expression	<1.00E-07	<1.00E-07	0.026	METABOLIC
A_23_P113111	AR	4.12	Higher expression	1.77E-05	3.18E-04	0.066	ESTROGEN_RESPONSE
A_33_P3339375	ARHGAP11B	-6.65	Lower expression	<1.00E-07	<1.00E-07	0.006	
A_33_P3234794	ARHGAP40	5.1	Higher expression	<1.00E-07	<1.00E-07	0.023	
A_33_P3254708	ARHGAP40	4.5	Higher expression	3.94E-06	9.39E-05	0.044	
A_23_P252276	ARHGEF38	6.21	Higher expression	<1.00E-07	<1.00E-07	0.01	
A_33_P3211864	ARMCX4	4.96	Higher expression	<1.00E-07	<1.00E-07	0.027	
A_23_P52017	ASPM	-4.92	Lower expression	<1.00E-07	<1.00E-07	0.029	WNT_BETA_CATENIN_SIGNALING
A_23_P216068	ATAD2	-5.55	Lower expression	<1.00E-07	<1.00E-07	0.017	CELL_CYCLE
A_33_P3214096	ATF3	4.4	Higher expression	3.94E-06	9.39E-05	0.05	
A_33_P3214105	ATF3	3.89	Higher expression	5.71E-05	8.16E-04	0.083	
A_23_P62932	ATP1B1	4.24	Higher expression	5.91E-06	1.19E-04	0.059	METABOLIC
A_23_P146943	ATP1B1	4.2	Higher expression	9.85E-06	1.86E-04	0.061	METABOLIC
A_23_P252322	ATP5E	-3.97	Lower expression	4.14E-05	6.47E-04	0.076	METABOLIC
A_23_P146058	ATP6V1C1	-5.36	Lower expression	<1.00E-07	<1.00E-07	0.019	METABOLIC

A_33_P3330952	ATP8A1	-3.97	Lower expression	4.53E-05	6.95E-04	0.076	
A_33_P3375314	ATP9A	-4	Lower expression	3.94E-05	6.27E-04	0.074	
A_23_P131866	AURKA	-6.22	Lower expression	<1.00E-07	<1.00E-07	0.009	CELL_CYCLE
A_23_P71270	AZGP1	5.88	Higher expression	<1.00E-07	<1.00E-07	0.012	ANDROGEN_RESPONSE
A_23_P37441	B2M	-5.18	Lower expression	<1.00E-07	<1.00E-07	0.021	IMMUNE
A_24_P143492	BCAS4	-4.36	Lower expression	3.94E-06	9.39E-05	0.052	
A_23_P118815	BIRC5	-8.38	Lower expression	<1.00E-07	<1.00E-07	0.001	CELL_CYCLE
A_24_P86240	BMP2K	3.88	Higher expression	5.91E-05	8.40E-04	0.083	METABOLIC
A_23_P134925	BNIP3L	3.88	Higher expression	6.11E-05	8.62E-04	0.083	APOPTOSIS
A_32_P53486	BOLA2B	-4.35	Lower expression	3.94E-06	9.39E-05	0.053	
A_23_P43800	BOP1	-5.37	Lower expression	<1.00E-07	<1.00E-07	0.018	
A_23_P71946	BSPRY	4	Higher expression	3.94E-05	6.27E-04	0.074	
A_23_P39465	BST2	-4.05	Lower expression	3.15E-05	5.33E-04	0.07	IMMUNE
A_23_P62901	BTG2	4.56	Higher expression	<1.00E-07	<1.00E-07	0.042	
A_33_P3344451	C16orf13	-4.03	Lower expression	3.35E-05	5.54E-04	0.072	
A_23_P206498	C16orf13	-3.88	Lower expression	6.11E-05	8.62E-04	0.083	
A_32_P182299	C1orf168	4.74	Higher expression	<1.00E-07	<1.00E-07	0.035	
A_23_P63243	C1orf43	-3.9	Lower expression	5.52E-05	7.98E-04	0.082	
A_24_P222655	C1QA	-4.61	Lower expression	<1.00E-07	<1.00E-07	0.04	IMMUNE
A_24_P194184	C20orf24	-4.73	Lower expression	<1.00E-07	<1.00E-07	0.035	
A_33_P3399433	C20orf27	-4.06	Lower expression	3.15E-05	5.33E-04	0.07	
A_33_P3289121	C2orf40	4.22	Higher expression	5.91E-06	1.19E-04	0.06	
A_33_P3249936	C3orf67	-4.82	Lower expression	<1.00E-07	<1.00E-07	0.032	
A_23_P42282	C4B	4.81	Higher expression	<1.00E-07	<1.00E-07	0.032	IMMUNE
A_23_P153562	C5AR1	-3.96	Lower expression	4.53E-05	6.95E-04	0.077	IMMUNE
A_23_P331235	C5orf38	5.85	Higher expression	<1.00E-07	<1.00E-07	0.013	
A_33_P3615922	C7orf63	5.19	Higher expression	<1.00E-07	<1.00E-07	0.021	
A_33_P3293202	C8orf37	-3.88	Lower expression	5.91E-05	8.40E-04	0.083	
A_33_P3422258	C8orf59	-4.92	Lower expression	<1.00E-07	<1.00E-07	0.029	
A_23_P9392	C9orf114	4.71	Higher expression	<1.00E-07	<1.00E-07	0.036	
A_23_P365767	CACNA1D	4.99	Higher expression	<1.00E-07	<1.00E-07	0.026	PROTEIN_SECRETION
A_24_P282210	CAPN15	-4.1	Lower expression	2.17E-05	3.81E-04	0.067	
A_23_P83381	CAPN8	6.45	Higher expression	<1.00E-07	<1.00E-07	0.008	
A_23_P94782	CAPN8	5.45	Higher expression	<1.00E-07	<1.00E-07	0.018	
A_24_P921086	CAPN8	4.73	Higher expression	<1.00E-07	<1.00E-07	0.036	
A_23_P201295	CASZ1	4.31	Higher expression	3.94E-06	9.39E-05	0.055	
A_33_P3338300	CASZ1	3.88	Higher expression	5.91E-05	8.40E-04	0.083	
A_33_P3423949	CBX2	-4.38	Lower expression	3.94E-06	9.39E-05	0.051	
A_23_P39574	CCDC150	-4.32	Lower expression	3.94E-06	9.39E-05	0.054	
A_33_P3260100	CCDC167	-5.82	Lower expression	<1.00E-07	<1.00E-07	0.013	
A_33_P3409337	CCDC176	4.47	Higher expression	3.94E-06	9.39E-05	0.046	
A_33_P3302518	CCDC40	4.38	Higher expression	3.94E-06	9.39E-05	0.051	
A_24_P61864	CCDC47	-4.62	Lower expression	<1.00E-07	<1.00E-07	0.039	
A_23_P152838	CCL5	-4.14	Lower expression	1.38E-05	2.52E-04	0.065	IMMUNE
A_23_P58321	CCNA2	-6.58	Lower expression	<1.00E-07	<1.00E-07	0.006	CELL_CYCLE
A_23_P65757	CCNB2	-6.52	Lower expression	<1.00E-07	<1.00E-07	0.007	CELL_CYCLE
A_33_P3385870	CCNG2	5.05	Higher expression	<1.00E-07	<1.00E-07	0.025	
A_24_P80532	CCNG2	4.34	Higher expression	3.94E-06	9.39E-05	0.053	

A_23_P72989	CCR4	-4.42	Lower expression	3.94E-06	9.39E-05	0.049	IMMUNE
A_23_P102404	CCT7	-5.11	Lower expression	<1.00E-07	<1.00E-07	0.023	MYC_TARGETS
A_23_P113572	CD19	-3.89	Lower expression	5.52E-05	7.98E-04	0.082	PI3K_AKT_MTOR
A_23_P85250	CD24	-4.4	Lower expression	3.94E-06	9.39E-05	0.05	ESTROGEN_RESPONSE
A_23_P6935	CD47	-4.85	Lower expression	<1.00E-07	<1.00E-07	0.031	IMMUNE
A_23_P74547	CD53	-4.46	Lower expression	3.94E-06	9.39E-05	0.047	
A_23_P15394	CD68	-4.23	Lower expression	5.91E-06	1.19E-04	0.059	
A_23_P361940	CD84	-3.94	Lower expression	4.93E-05	7.43E-04	0.078	
A_24_P409042	CDC42SE2	-4.21	Lower expression	5.91E-06	1.19E-04	0.061	IMMUNE
A_23_P104651	CDCA5	-4.93	Lower expression	<1.00E-07	<1.00E-07	0.028	
A_23_P138507	CDK1	-7	Lower expression	<1.00E-07	<1.00E-07	0.004	CELL_CYCLE
A_33_P3341239	CDK16	-3.9	Lower expression	5.52E-05	7.98E-04	0.081	PROTEIN_SECRETION
A_33_P3226985	CDK5R1	-4.27	Lower expression	5.91E-06	1.19E-04	0.057	P53_PATHWAY
A_33_P3386262	CDT1	-4.95	Lower expression	<1.00E-07	<1.00E-07	0.028	CELL_CYCLE
A_23_P401	CENPF	-5.77	Lower expression	<1.00E-07	<1.00E-07	0.014	CELL_CYCLE
A_24_P419132	CENPI	-4.01	Lower expression	3.94E-05	6.27E-04	0.074	
A_33_P3387831	CENPM	-5.98	Lower expression	<1.00E-07	<1.00E-07	0.011	CELL_CYCLE
A_23_P115872	CEP55	-4.94	Lower expression	<1.00E-07	<1.00E-07	0.028	
A_23_P163306	CGNL1	6.42	Higher expression	<1.00E-07	<1.00E-07	0.008	
A_24_P400376	CHCHD2	-4.05	Lower expression	3.35E-05	5.54E-04	0.071	HYPOXIA
A_23_P105571	CHPT1	4.17	Higher expression	9.85E-06	1.86E-04	0.063	ESTROGEN_RESPONSE
A_23_P151895	CILP	4.01	Higher expression	3.74E-05	6.06E-04	0.073	
A_23_P35467	CISD1	-3.9	Lower expression	5.52E-05	7.98E-04	0.081	
A_33_P3362641	CISD2	-4.19	Lower expression	9.85E-06	1.86E-04	0.062	
A_23_P163235	CKMT1A	-4.26	Lower expression	5.91E-06	1.19E-04	0.058	
A_23_P29800	CLDN11	4.54	Higher expression	1.97E-06	5.50E-05	0.042	EPITHELIAL_STRUCTURE
A_33_P3285565	CLDN3	-4.39	Lower expression	3.94E-06	9.39E-05	0.051	METABOLIC
A_23_P106544	CMC2	-4.02	Lower expression	3.55E-05	5.78E-04	0.072	
A_23_P11295	CMC4	-4.04	Lower expression	3.35E-05	5.54E-04	0.072	
A_33_P3401826	CMPK2	-4.15	Lower expression	1.38E-05	2.52E-04	0.064	
A_23_P124946	CMYA5	4.75	Higher expression	<1.00E-07	<1.00E-07	0.035	
A_23_P200507	CNIH4	-5.09	Lower expression	<1.00E-07	<1.00E-07	0.024	
A_33_P3422728	CNTNAP3	3.99	Higher expression	4.14E-05	6.47E-04	0.075	
A_32_P80850	COL14A1	4.47	Higher expression	3.94E-06	9.39E-05	0.046	
A_23_P160318	COL16A1	4.61	Higher expression	<1.00E-07	<1.00E-07	0.04	EPITHELIAL_STRUCTURE
A_23_P45786	COL9A2	4.04	Higher expression	3.35E-05	5.54E-04	0.071	
A_33_P3257222	COMTD1	-4.14	Lower expression	1.38E-05	2.52E-04	0.065	
A_23_P44617	COPG1	-4.27	Lower expression	3.94E-06	9.39E-05	0.056	
A_23_P65157	COX17	-4.98	Lower expression	<1.00E-07	<1.00E-07	0.026	
A_23_P402751	COX2	-5.18	Lower expression	<1.00E-07	<1.00E-07	0.022	
A_23_P115064	CRABP2	-4.1	Lower expression	2.17E-05	3.81E-04	0.067	METABOLIC
A_33_P3280531	CRAT	4.55	Higher expression	1.97E-06	5.50E-05	0.042	METABOLIC
A_23_P502575	CSNK2A1	-4.44	Lower expression	3.94E-06	9.39E-05	0.048	
A_32_P109242	CSRNP3	4.56	Higher expression	<1.00E-07	<1.00E-07	0.042	APOPTOSIS
A_33_P3420043	CSRNP3	4.22	Higher expression	5.91E-06	1.19E-04	0.06	APOPTOSIS
A_23_P501435	CSRP2BP	-3.88	Lower expression	5.91E-05	8.40E-04	0.083	CELL_CYCLE
A_24_P256552	CSTF3	-4.29	Lower expression	3.94E-06	9.39E-05	0.056	DNA_REPAIR
A_33_P3401556	CTLA4	-5.16	Lower expression	<1.00E-07	<1.00E-07	0.022	IMMUNE

A_23_P46141	CTSS	-3.87	Lower expression	6.50E-05	9.13E-04	0.084	
A_23_P407565	CX3CR1	4.5	Higher expression	3.94E-06	9.39E-05	0.044	
A_33_P3343175	CXCL10	-4.6	Lower expression	<1.00E-07	<1.00E-07	0.04	IMMUNE
A_24_P303091	CXCL10	-4.09	Lower expression	2.36E-05	4.11E-04	0.068	IMMUNE
A_24_P20607	CXCL11	-4.52	Lower expression	3.94E-06	9.39E-05	0.044	IMMUNE
A_23_P18452	CXCL9	-5.6	Lower expression	<1.00E-07	<1.00E-07	0.016	IMMUNE
A_23_P102000	CXCR4	-4.08	Lower expression	2.56E-05	4.42E-04	0.068	
A_23_P399001	CXXC5	-4.98	Lower expression	<1.00E-07	<1.00E-07	0.026	
A_24_P228026	CYB5D2	3.92	Higher expression	5.32E-05	7.84E-04	0.08	
A_24_P345451	CYBRD1	7.18	Higher expression	<1.00E-07	<1.00E-07	0.003	
A_23_P209564	CYBRD1	7.14	Higher expression	<1.00E-07	<1.00E-07	0.003	
A_24_P573978	CYCS	-4.62	Lower expression	<1.00E-07	<1.00E-07	0.039	METABOLIC
A_24_P89843	CYHR1	-3.94	Lower expression	5.12E-05	7.64E-04	0.079	
A_24_P293530	CYP4X1	6.53	Higher expression	<1.00E-07	<1.00E-07	0.007	
A_33_P3279880	CYP4Z1	7.32	Higher expression	<1.00E-07	<1.00E-07	0.003	
A_23_P103971	CYP4Z1	6.67	Higher expression	<1.00E-07	<1.00E-07	0.006	
A_24_P145529	CYP4Z2P	6.62	Higher expression	<1.00E-07	<1.00E-07	0.006	
A_32_P6769	D2HGDH	4.11	Higher expression	1.97E-05	3.50E-04	0.066	METABOLIC
A_23_P215875	DCAF13	-3.97	Lower expression	4.53E-05	6.95E-04	0.077	
A_33_P3322307	DDX11	-4.11	Lower expression	2.17E-05	3.81E-04	0.067	
A_23_P57534	DDX17	4.54	Higher expression	3.94E-06	9.39E-05	0.043	ESTROGEN_RESPONSE
A_23_P30069	DDX60L	-4.27	Lower expression	5.91E-06	1.19E-04	0.057	
A_23_P330908	DERL1	-5.62	Lower expression	<1.00E-07	<1.00E-07	0.016	
A_33_P3271990	DGAT1	-4.75	Lower expression	<1.00E-07	<1.00E-07	0.034	
A_24_P343095	DHFR	-5.76	Lower expression	<1.00E-07	<1.00E-07	0.014	PI3K_AKT_MTOR
A_32_P211045	DHFR	-4.01	Lower expression	3.74E-05	6.06E-04	0.073	PI3K_AKT_MTOR
A_23_P395426	DIDO1	-4.33	Lower expression	3.94E-06	9.39E-05	0.054	APOPTOSIS
A_23_P149121	DIRAS3	5.82	Higher expression	<1.00E-07	<1.00E-07	0.013	
A_33_P3399090	DIXDC1	3.9	Higher expression	5.52E-05	7.98E-04	0.082	WNT_BETA_CATENIN_SIGNALING
A_23_P28598	DLX2	6.72	Higher expression	<1.00E-07	<1.00E-07	0.005	HOMEOBOX
A_24_P205316	DNAJC5	-5.05	Lower expression	<1.00E-07	<1.00E-07	0.025	APOPTOSIS
A_33_P3363153	DNAJC5	-4.09	Lower expression	2.36E-05	4.11E-04	0.068	APOPTOSIS
A_23_P10385	DTL	-6.36	Lower expression	<1.00E-07	<1.00E-07	0.009	DNA_REPAIR
A_23_P88848	DUS2	-4.71	Lower expression	<1.00E-07	<1.00E-07	0.037	
A_23_P110712	DUSP1	4	Higher expression	3.94E-05	6.27E-04	0.074	IMMUNE
A_23_P134935	DUSP4	4.97	Higher expression	<1.00E-07	<1.00E-07	0.027	IMMUNE
A_23_P154108	DYNC1I2	6.41	Higher expression	<1.00E-07	<1.00E-07	0.008	CELL_CYCLE
A_23_P408955	E2F2	-6.05	Lower expression	<1.00E-07	<1.00E-07	0.011	
A_32_P210202	E2F7	-6.58	Lower expression	<1.00E-07	<1.00E-07	0.006	
A_23_P35871	E2F8	-5.66	Lower expression	<1.00E-07	<1.00E-07	0.015	CELL_CYCLE
A_32_P15320	EEF1A1	4.01	Higher expression	3.74E-05	6.06E-04	0.073	
A_23_P432545	EFCAB4A	5.34	Higher expression	<1.00E-07	<1.00E-07	0.019	
A_23_P34710	EGLN1	-4.87	Lower expression	<1.00E-07	<1.00E-07	0.03	REACTIVE_OXYGEN_SPECIES_PATHWAY
A_33_P3411744	EGOT	4.63	Higher expression	<1.00E-07	<1.00E-07	0.039	
A_23_P214080	EGR1	4.39	Higher expression	3.94E-06	9.39E-05	0.05	IMMUNE
A_23_P216225	EGR3	4.3	Higher expression	3.94E-06	9.39E-05	0.055	APOPTOSIS
A_33_P3412563	EIF3IP1	4	Higher expression	3.94E-05	6.27E-04	0.074	

A_33_P3385002	ELK1	-4.15	Lower expression	1.38E-05	2.52E-04	0.064	APOPTOSIS
A_23_P210538	ELMO2	-4.36	Lower expression	3.94E-06	9.39E-05	0.052	APOPTOSIS
A_24_P322635	ELMO2	-3.95	Lower expression	4.53E-05	6.95E-04	0.077	APOPTOSIS
A_23_P215454	ELN	4.5	Higher expression	3.94E-06	9.39E-05	0.044	EPITHELIAL_MESENCHYMAL_TRANSITION
A_23_P156497	ELOVL5	6.02	Higher expression	<1.00E-07	<1.00E-07	0.011	METABOLIC
A_23_P60002	EMC2	-4.21	Lower expression	5.91E-06	1.19E-04	0.061	
A_23_P82748	ENY2	-4.08	Lower expression	2.56E-05	4.42E-04	0.069	PROTEIN_SECRETION
A_24_P257579	EPB41L4A	3.93	Higher expression	5.12E-05	7.64E-04	0.079	
A_24_P943205	EPSTI1	-4.82	Lower expression	<1.00E-07	<1.00E-07	0.032	IMMUNE
A_33_P3213645	ERN2	-7.38	Lower expression	<1.00E-07	<1.00E-07	0.002	APOPTOSIS
A_23_P32707	ESPL1	-7.59	Lower expression	<1.00E-07	<1.00E-07	0.002	CELL_CYCLE
A_24_P7965	ESRRG	5.39	Higher expression	<1.00E-07	<1.00E-07	0.018	
A_33_P3210343	ETV6	4.26	Higher expression	5.91E-06	1.19E-04	0.058	
A_23_P140427	EVL	4.06	Higher expression	3.15E-05	5.33E-04	0.07	EPITHELIAL_STRUCTURE
A_33_P3252196	EZH2	-5.71	Lower expression	<1.00E-07	<1.00E-07	0.015	CELL_CYCLE
A_33_P3226832	F3	4.18	Higher expression	9.85E-06	1.86E-04	0.062	IMMUNE
A_23_P86917	FADD	-4.31	Lower expression	3.94E-06	9.39E-05	0.055	APOPTOSIS
A_24_P192994	FADS1	-4.33	Lower expression	3.94E-06	9.39E-05	0.054	METABOLIC
A_23_P371966	FAM171B	4.68	Higher expression	<1.00E-07	<1.00E-07	0.037	
A_24_P111912	FAM172A	4.41	Higher expression	3.94E-06	9.39E-05	0.05	
A_23_P422831	FAM189A2	4.05	Higher expression	3.35E-05	5.54E-04	0.071	
A_23_P63660	FAM213A	-3.99	Lower expression	3.94E-05	6.27E-04	0.075	
A_33_P3279124	FAM21C	4.06	Higher expression	3.15E-05	5.33E-04	0.069	
A_23_P323751	FAM83D	-6.37	Lower expression	<1.00E-07	<1.00E-07	0.008	
A_33_P3416037	FAM96A	-4	Lower expression	3.94E-05	6.27E-04	0.074	
A_33_P3332492	FANK1	6.64	Higher expression	<1.00E-07	<1.00E-07	0.006	
A_23_P115785	FANK1	5.77	Higher expression	<1.00E-07	<1.00E-07	0.014	
A_23_P63390	FCGR1B	-4.36	Lower expression	3.94E-06	9.39E-05	0.052	IMMUNE
A_23_P126298	FCGR3B	-5.32	Lower expression	<1.00E-07	<1.00E-07	0.019	
A_23_P80048	FER1L4	-3.99	Lower expression	4.14E-05	6.47E-04	0.075	
A_32_P71788	FKBP4	-4.37	Lower expression	3.94E-06	9.39E-05	0.051	ESTROGEN_RESPONSE
A_33_P3376214	FMO5	5.37	Higher expression	<1.00E-07	<1.00E-07	0.019	
A_23_P233	FMO5	5.24	Higher expression	<1.00E-07	<1.00E-07	0.02	
A_24_P40417	FMR1	-5.18	Lower expression	<1.00E-07	<1.00E-07	0.021	
A_23_P106194	FOS	5.71	Higher expression	<1.00E-07	<1.00E-07	0.015	IMMUNE
A_23_P429998	FOSB	5.18	Higher expression	<1.00E-07	<1.00E-07	0.021	IMMUNE
A_33_P3223503	FRY	4.9	Higher expression	<1.00E-07	<1.00E-07	0.029	
A_23_P50504	FTL	-5.27	Lower expression	<1.00E-07	<1.00E-07	0.02	REACTIVE_OXYGEN_SPECIES_PATHWAY
A_33_P3272580	FUCA2	-6.17	Lower expression	<1.00E-07	<1.00E-07	0.01	
A_23_P328545	GABRP	4.42	Higher expression	3.94E-06	9.39E-05	0.049	
A_23_P13899	GAPDH	-3.87	Lower expression	6.70E-05	9.37E-04	0.084	PI3K_AKT_MTOR
A_23_P131020	GATAD2A	-3.93	Lower expression	5.12E-05	7.64E-04	0.079	
A_24_P167642	GCH1	-3.88	Lower expression	5.91E-05	8.40E-04	0.083	IMMUNE
A_33_P3282489	GCNT1	-4.73	Lower expression	<1.00E-07	<1.00E-07	0.035	IMMUNE
A_33_P3340025	GINS1	-7.25	Lower expression	<1.00E-07	<1.00E-07	0.003	CELL_CYCLE
A_23_P152136	GINS3	-4.55	Lower expression	1.97E-06	5.50E-05	0.042	CELL_CYCLE
A_33_P3390102	GIPC1	-3.94	Lower expression	4.93E-05	7.43E-04	0.078	IMMUNE

A_23_P151870	GLCE	4.49	Higher expression	3.94E-06	9.39E-05	0.045	METABOLIC
A_33_P3405334	GM2A	-4.53	Lower expression	3.94E-06	9.39E-05	0.043	P53_PATHWAY
A_24_P941441	GNA13	-4.71	Lower expression	<1.00E-07	<1.00E-07	0.037	ANGIOGENESIS
A_24_P190007	GP2	4.62	Higher expression	<1.00E-07	<1.00E-07	0.039	KRAS_SIGNALING
A_23_P435407	GPC4	4.22	Higher expression	5.91E-06	1.19E-04	0.06	METABOLIC
A_23_P343382	GPRIN2	4.02	Higher expression	3.55E-05	5.78E-04	0.072	
A_23_P305977	GRAMD2	4.8	Higher expression	<1.00E-07	<1.00E-07	0.032	
A_23_P119040	GREB1L	5.47	Higher expression	<1.00E-07	<1.00E-07	0.017	
A_33_P3353471	GRIK1-AS1	6.82	Higher expression	<1.00E-07	<1.00E-07	0.005	
A_23_P316960	GRINA	-4.42	Lower expression	3.94E-06	9.39E-05	0.048	UV_RESPONSE
A_23_P152420	GSE1	-5.1	Lower expression	<1.00E-07	<1.00E-07	0.023	
A_33_P3423365	GSN	4.18	Higher expression	9.85E-06	1.86E-04	0.063	APOPTOSIS
A_23_P397208	GSTM2	6.16	Higher expression	<1.00E-07	<1.00E-07	0.01	METABOLIC
A_33_P3410351	GSTM2	5.46	Higher expression	<1.00E-07	<1.00E-07	0.018	METABOLIC
A_23_P58869	GSTM2P1	5.67	Higher expression	<1.00E-07	<1.00E-07	0.015	
A_23_P111621	GTF2IRD1	-4.21	Lower expression	5.91E-06	1.19E-04	0.061	
A_33_P3239455	GTF2IRD2B	3.95	Higher expression	4.53E-05	6.95E-04	0.078	
A_23_P57588	GTSE1	-4.98	Lower expression	<1.00E-07	<1.00E-07	0.027	
A_33_P3269109	GUCY1A3	4.14	Higher expression	1.38E-05	2.52E-04	0.065	KRAS_SIGNALING
A_24_P336441	GUK1	-4.16	Lower expression	9.85E-06	1.86E-04	0.063	DNA_REPAIR
A_24_P38895	H2AFX	-5.46	Lower expression	<1.00E-07	<1.00E-07	0.018	CELL_CYCLE
A_24_P75190	HBD	4.01	Higher expression	3.74E-05	6.06E-04	0.073	METABOLIC
A_33_P3258117	HELLS	-6.63	Lower expression	<1.00E-07	<1.00E-07	0.006	CELL_CYCLE
A_23_P12816	HELLS	-4.75	Lower expression	<1.00E-07	<1.00E-07	0.034	CELL_CYCLE
A_33_P3400374	HELZ2	-4.29	Lower expression	3.94E-06	9.39E-05	0.056	
A_32_P211080	HERC2P2	3.93	Higher expression	5.12E-05	7.64E-04	0.079	
A_33_P3265359	HES6	-5.23	Lower expression	<1.00E-07	<1.00E-07	0.02	METABOLIC
A_23_P250385	HIST1H1B	-5.09	Lower expression	<1.00E-07	<1.00E-07	0.024	HISTONE
A_23_P122443	HIST1H1C	-4.18	Lower expression	9.85E-06	1.86E-04	0.062	HISTONE
A_23_P428184	HIST1H2AD	-5.93	Lower expression	<1.00E-07	<1.00E-07	0.012	HISTONE
A_24_P303354	HIST1H2AG	-5.87	Lower expression	<1.00E-07	<1.00E-07	0.012	HISTONE
A_23_P81859	HIST1H2AH	-5.98	Lower expression	<1.00E-07	<1.00E-07	0.011	HISTONE
A_33_P3360216	HIST1H2AI	-7.56	Lower expression	<1.00E-07	<1.00E-07	0.002	HISTONE
A_33_P3344086	HIST1H2AJ	-6.46	Lower expression	<1.00E-07	<1.00E-07	0.007	HISTONE
A_24_P217848	HIST1H2AK	-5.87	Lower expression	<1.00E-07	<1.00E-07	0.012	HISTONE
A_23_P363174	HIST1H2AL	-6.55	Lower expression	<1.00E-07	<1.00E-07	0.007	HISTONE
A_32_P221799	HIST1H2AM	-5.72	Lower expression	<1.00E-07	<1.00E-07	0.014	HISTONE
A_24_P86389	HIST1H2AM	-5.09	Lower expression	<1.00E-07	<1.00E-07	0.023	HISTONE
A_23_P111054	HIST1H2BB	-5.79	Lower expression	<1.00E-07	<1.00E-07	0.013	HISTONE
A_23_P93180	HIST1H2BC	-4.57	Lower expression	<1.00E-07	<1.00E-07	0.041	HISTONE
A_33_P3229122	HIST1H2BF	-5.09	Lower expression	<1.00E-07	<1.00E-07	0.023	HISTONE
A_23_P167997	HIST1H2BG	-4.46	Lower expression	3.94E-06	9.39E-05	0.046	HISTONE
A_23_P366216	HIST1H2BH	-5.7	Lower expression	<1.00E-07	<1.00E-07	0.015	HISTONE
A_23_P111041	HIST1H2BI	-5.74	Lower expression	<1.00E-07	<1.00E-07	0.014	HISTONE
A_33_P3229083	HIST1H2BK	-4.21	Lower expression	5.91E-06	1.19E-04	0.061	HISTONE
A_23_P8013	HIST1H2BL	-5.48	Lower expression	<1.00E-07	<1.00E-07	0.017	HISTONE
A_24_P3783	HIST1H2BM	-6.12	Lower expression	<1.00E-07	<1.00E-07	0.01	HISTONE
A_23_P59069	HIST1H2BO	-5.7	Lower expression	<1.00E-07	<1.00E-07	0.015	HISTONE

A_23_P93258	HIST1H3B	-8.77	Lower expression	<1.00E-07	<1.00E-07	<0.001	HISTONE
A_23_P133814	HIST1H3C	-5.83	Lower expression	<1.00E-07	<1.00E-07	0.013	HISTONE
A_24_P217834	HIST1H3D	-6.08	Lower expression	<1.00E-07	<1.00E-07	0.01	HISTONE
A_23_P70445	HIST1H3E	-5.97	Lower expression	<1.00E-07	<1.00E-07	0.011	HISTONE
A_23_P30799	HIST1H3F	-7.11	Lower expression	<1.00E-07	<1.00E-07	0.003	HISTONE
A_23_P42198	HIST1H3G	-7.91	Lower expression	<1.00E-07	<1.00E-07	0.001	HISTONE
A_33_P3404989	HIST1H3H	-8.02	Lower expression	<1.00E-07	<1.00E-07	0.001	HISTONE
A_33_P3287879	HIST1H3H	-7.66	Lower expression	<1.00E-07	<1.00E-07	0.002	HISTONE
A_24_P9321	HIST1H3I	-4.81	Lower expression	<1.00E-07	<1.00E-07	0.032	HISTONE
A_23_P93282	HIST1H3J	-4.48	Lower expression	3.94E-06	9.39E-05	0.046	HISTONE
A_23_P431179	HIST1H4A	-7.04	Lower expression	<1.00E-07	<1.00E-07	0.004	HISTONE
A_23_P214487	HIST1H4C	-4.46	Lower expression	3.94E-06	9.39E-05	0.047	HISTONE
A_23_P395374	HIST1H4D	-5.18	Lower expression	<1.00E-07	<1.00E-07	0.021	HISTONE
A_23_P359540	HIST1H4F	-6.96	Lower expression	<1.00E-07	<1.00E-07	0.004	HISTONE
A_23_P323685	HIST1H4H	-6.33	Lower expression	<1.00E-07	<1.00E-07	0.009	HISTONE
A_24_P20873	HIST1H4I	-5.01	Lower expression	<1.00E-07	<1.00E-07	0.025	HISTONE
A_33_P3299865	HIST1H4K	-5.32	Lower expression	<1.00E-07	<1.00E-07	0.019	HISTONE
A_23_P30813	HIST1H4K	-4.46	Lower expression	3.94E-06	9.39E-05	0.047	HISTONE
A_33_P3351851	HIST1H4L	-4.96	Lower expression	<1.00E-07	<1.00E-07	0.027	HISTONE
A_23_P309381	HIST2H2AA4	-4.65	Lower expression	<1.00E-07	<1.00E-07	0.038	HISTONE
A_33_P3257678	HIST2H3A	-9.81	Lower expression	<1.00E-07	<1.00E-07	<0.001	HISTONE
A_24_P324465	HIST2H3D	-8.54	Lower expression	<1.00E-07	<1.00E-07	0.001	HISTONE
A_23_P115375	HIST2H3D	-7.1	Lower expression	<1.00E-07	<1.00E-07	0.003	HISTONE
A_23_P436281	HIST2H4B	-6.39	Lower expression	<1.00E-07	<1.00E-07	0.008	HISTONE
A_33_P3807062	HJURP	-7.42	Lower expression	<1.00E-07	<1.00E-07	0.002	
A_33_P3379962	HLA-A	-4.22	Lower expression	5.91E-06	1.19E-04	0.06	IMMUNE
A_33_P3400578	HLF	4.03	Higher expression	3.35E-05	5.54E-04	0.072	
A_23_P155765	HMGB2	-4.27	Lower expression	5.91E-06	1.19E-04	0.056	APOPTOSIS
A_33_P3237359	HMGB3	-5.38	Lower expression	<1.00E-07	<1.00E-07	0.018	CELL_CYCLE
A_33_P3319041	HMGB3	-5.45	Lower expression	<1.00E-07	<1.00E-07	0.018	CELL_CYCLE
A_33_P3318414	HMHA1	-4.17	Lower expression	9.85E-06	1.86E-04	0.063	
A_23_P100632	HN1	-4.78	Lower expression	<1.00E-07	<1.00E-07	0.033	CELL_CYCLE
A_23_P434900	HN1L	-4.12	Lower expression	1.77E-05	3.18E-04	0.066	
A_24_P829209	HOXA-AS3	4.27	Higher expression	3.94E-06	9.39E-05	0.056	HOMEOBOX
A_23_P93772	HOXA5	4.58	Higher expression	<1.00E-07	<1.00E-07	0.041	HOMEOBOX
A_23_P70968	HOXA7	5.18	Higher expression	<1.00E-07	<1.00E-07	0.021	HOMEOBOX
A_33_P3416231	HOXA9	4.41	Higher expression	3.94E-06	9.39E-05	0.049	HOMEOBOX
A_23_P500998	HOXA9	4.04	Higher expression	3.35E-05	5.54E-04	0.071	HOMEOBOX
A_23_P316511	HOXB3	5.54	Higher expression	<1.00E-07	<1.00E-07	0.017	HOMEOBOX
A_24_P416370	HOXB4	4.76	Higher expression	<1.00E-07	<1.00E-07	0.034	HOMEOBOX
A_23_P47941	HOXC11	-4.27	Lower expression	5.91E-06	1.19E-04	0.057	HOMEOBOX
A_23_P134714	HRSP12	-4.5	Lower expression	3.94E-06	9.39E-05	0.044	
A_32_P53183	HSD17B7	4.33	Higher expression	3.94E-06	9.39E-05	0.054	METABOLIC
A_23_P253841	HSF1	-4.1	Lower expression	2.17E-05	3.81E-04	0.067	
A_23_P162874	HSP90AA1	-4.5	Lower expression	3.94E-06	9.39E-05	0.044	METABOLIC
A_33_P3665777	HSP90AA1	-4.37	Lower expression	3.94E-06	9.39E-05	0.052	METABOLIC
A_23_P145089	HSP90AB1	-5.16	Lower expression	<1.00E-07	<1.00E-07	0.022	MYC_TARGETS
A_24_P823684	HSP90AB1	-3.97	Lower expression	4.53E-05	6.95E-04	0.076	MYC_TARGETS

A_24_P161525	HSP90AB3P	-4.07	Lower expression	2.76E-05	4.72E-04	0.069	
A_33_P3287646	HSPB1	-7.04	Lower expression	<1.00E-07	<1.00E-07	0.004	APOPTOSIS
A_23_P162579	HSPB8	-4.26	Lower expression	5.91E-06	1.19E-04	0.057	ESTROGEN_RESPONSE
A_32_P25273	HSPD1	-3.99	Lower expression	4.14E-05	6.47E-04	0.076	
A_23_P56922	HSPE1	-4.79	Lower expression	<1.00E-07	<1.00E-07	0.033	
A_23_P64129	HTATIP2	-4.88	Lower expression	<1.00E-07	<1.00E-07	0.03	METABOLIC
A_23_P117683	HYPK	-4.43	Lower expression	3.94E-06	9.39E-05	0.048	
A_32_P36235	IER2	4.12	Higher expression	1.77E-05	3.18E-04	0.066	IMMUNE
A_24_P270460	IFI27	-5.56	Lower expression	<1.00E-07	<1.00E-07	0.016	IMMUNE
A_23_P23074	IFI44	-4.45	Lower expression	3.94E-06	9.39E-05	0.047	IMMUNE
A_23_P45870	IFI44L	-5.08	Lower expression	<1.00E-07	<1.00E-07	0.024	
A_23_P45871	IFI44L	-4.08	Lower expression	2.56E-05	4.42E-04	0.068	
A_23_P52266	IFIT1	-5.01	Lower expression	<1.00E-07	<1.00E-07	0.025	IMMUNE
A_24_P304071	IFIT2	-3.95	Lower expression	4.53E-05	6.95E-04	0.078	IMMUNE
A_24_P30194	IFIT5	-4.23	Lower expression	5.91E-06	1.19E-04	0.059	
A_23_P116207	IFT46	4.78	Higher expression	<1.00E-07	<1.00E-07	0.033	
A_23_P421379	IGF2	4.77	Higher expression	<1.00E-07	<1.00E-07	0.033	KRAS_SIGNALING
A_32_P78101	IGSF21	5.23	Higher expression	<1.00E-07	<1.00E-07	0.02	
A_23_P203173	IL10RA	-3.99	Lower expression	4.14E-05	6.47E-04	0.075	
A_24_P203000	IL2RB	-3.91	Lower expression	5.32E-05	7.84E-04	0.08	
A_23_P148473	IL2RG	-4.72	Lower expression	<1.00E-07	<1.00E-07	0.036	
A_23_P404494	IL7R	-4.7	Lower expression	<1.00E-07	<1.00E-07	0.037	
A_23_P50081	IMPA2	-4.92	Lower expression	<1.00E-07	<1.00E-07	0.029	ESTROGEN_RESPONSE
A_24_P942454	INADL	6.21	Higher expression	<1.00E-07	<1.00E-07	0.01	EPITHELIAL_STRUCTURE
A_23_P321034	INADL	3.87	Higher expression	6.70E-05	9.37E-04	0.084	EPITHELIAL_STRUCTURE
A_23_P116387	INCENP	-4.82	Lower expression	<1.00E-07	<1.00E-07	0.032	CELL_CYCLE
A_33_P3283083	INPP4B	4.25	Higher expression	5.91E-06	1.19E-04	0.058	
A_33_P3321342	INSIG2	-4.86	Lower expression	<1.00E-07	<1.00E-07	0.031	METABOLIC
A_23_P81087	INTS12	5.12	Higher expression	<1.00E-07	<1.00E-07	0.022	
A_23_P6836	IP6K2	4.67	Higher expression	<1.00E-07	<1.00E-07	0.037	P53_PATHWAY
A_33_P3250173	IQCJ	4.75	Higher expression	<1.00E-07	<1.00E-07	0.035	
A_33_P3250178	IQCJ	4.15	Higher expression	9.85E-06	1.86E-04	0.064	
A_33_P3321293	IQGAP3	-7	Lower expression	<1.00E-07	<1.00E-07	0.004	CELL_CYCLE
A_23_P162300	IRAK3	3.92	Higher expression	5.32E-05	7.84E-04	0.08	IMMUNE
A_33_P3343120	IRF8	-3.89	Lower expression	5.52E-05	7.98E-04	0.082	IMMUNE
A_23_P65442	IRF9	-5.09	Lower expression	<1.00E-07	<1.00E-07	0.023	IMMUNE
A_33_P3297562	IRX2	4.88	Higher expression	<1.00E-07	<1.00E-07	0.03	HOMEOBOX
A_23_P156025	IRX2	4.84	Higher expression	<1.00E-07	<1.00E-07	0.031	HOMEOBOX
A_23_P32404	ISG20	-4.98	Lower expression	<1.00E-07	<1.00E-07	0.026	ESTROGEN_RESPONSE
A_33_P3263432	ITGA10	3.9	Higher expression	5.52E-05	7.98E-04	0.081	EPITHELIAL_STRUCTURE
A_23_P329573	ITGB2	-4.07	Lower expression	2.76E-05	4.72E-04	0.069	IMMUNE
A_23_P109881	ITIH4	4.42	Higher expression	3.94E-06	9.39E-05	0.049	METABOLIC
A_24_P241815	JUNB	4.11	Higher expression	1.97E-05	3.50E-04	0.067	
A_24_P151	KCNAB2	-4.67	Lower expression	<1.00E-07	<1.00E-07	0.038	
A_33_P3319920	KDM4B	4.87	Higher expression	<1.00E-07	<1.00E-07	0.03	ESTROGEN_RESPONSE
A_33_P3396600	KDM4B	3.98	Higher expression	4.14E-05	6.47E-04	0.076	ESTROGEN_RESPONSE
A_23_P117852	KIAA0101	-7.04	Lower expression	<1.00E-07	<1.00E-07	0.004	DNA_REPAIR
A_24_P762886	KIAA0485	4.3	Higher expression	3.94E-06	9.39E-05	0.055	

A_32_P58614	KIAA1377	4.01	Higher expression	3.94E-05	6.27E-04	0.074	CELL_CYCLE
A_23_P410965	KIAA1522	4.23	Higher expression	5.91E-06	1.19E-04	0.059	
A_33_P3244083	KIF13B	7.88	Higher expression	<1.00E-07	<1.00E-07	0.001	P53_PATHWAY
A_23_P147388	KIF13B	6.22	Higher expression	<1.00E-07	<1.00E-07	0.009	P53_PATHWAY
A_32_P165611	KIF13B	5.6	Higher expression	<1.00E-07	<1.00E-07	0.016	P53_PATHWAY
A_33_P3230548	KIF14	-6.79	Lower expression	<1.00E-07	<1.00E-07	0.005	
A_33_P3311755	KIF23	-7.66	Lower expression	<1.00E-07	<1.00E-07	0.001	CELL_CYCLE
A_23_P254091	KIF2A	-5.17	Lower expression	<1.00E-07	<1.00E-07	0.022	
A_32_P154473	KIF5C	7.09	Higher expression	<1.00E-07	<1.00E-07	0.003	KRAS_SIGNALING
A_23_P133956	KIFC1	-6.57	Lower expression	<1.00E-07	<1.00E-07	0.006	CELL_CYCLE
A_23_P22232	KLRC2	-4.48	Lower expression	3.94E-06	9.39E-05	0.046	
A_32_P33263	KLRG2	-4.85	Lower expression	<1.00E-07	<1.00E-07	0.031	
A_33_P3224105	KNSTRN	-4.2	Lower expression	9.85E-06	1.86E-04	0.061	
A_24_P265346	KRT14	4.07	Higher expression	2.76E-05	4.72E-04	0.069	EPITHELIAL_STRUCTURE
A_23_P27133	KRT15	4.04	Higher expression	3.35E-05	5.54E-04	0.072	ESTROGEN_RESPONSE
A_24_P331704	KRT80	-4.83	Lower expression	<1.00E-07	<1.00E-07	0.031	
A_23_P73763	LAGE3	-4.11	Lower expression	1.97E-05	3.50E-04	0.067	
A_33_P3223780	LAMB2	3.95	Higher expression	4.53E-05	6.95E-04	0.078	
A_23_P86012	LAMB3	4.54	Higher expression	1.97E-06	5.50E-05	0.043	EPITHELIAL_STRUCTURE
A_23_P201636	LAMC2	4.81	Higher expression	<1.00E-07	<1.00E-07	0.032	EPITHELIAL_STRUCTURE
A_33_P3410409	LAMP2	-4.95	Lower expression	<1.00E-07	<1.00E-07	0.028	
A_23_P40295	LAMP5	4.48	Higher expression	3.94E-06	9.39E-05	0.046	
A_24_P180680	LAPTM4B	-5.31	Lower expression	<1.00E-07	<1.00E-07	0.019	
A_24_P414999	LAPTM4B	-4.96	Lower expression	<1.00E-07	<1.00E-07	0.027	
A_23_P30547	LCP2	-4.27	Lower expression	5.91E-06	1.19E-04	0.057	IMMUNE
A_23_P208493	LILRB2	-4.91	Lower expression	<1.00E-07	<1.00E-07	0.029	
A_32_P70158	LILRB3	-3.93	Lower expression	5.12E-05	7.64E-04	0.079	
A_23_P83403	LIMCH1	4.99	Higher expression	<1.00E-07	<1.00E-07	0.025	
A_32_P117354	LIMCH1	4.24	Higher expression	5.91E-06	1.19E-04	0.058	
A_33_P3312384	LINC00478	4.23	Higher expression	5.91E-06	1.19E-04	0.059	
A_33_P3245480	LINC00869	4.36	Higher expression	3.94E-06	9.39E-05	0.052	
A_24_P112941	LINGO1	-4.36	Lower expression	3.94E-06	9.39E-05	0.052	
A_23_P258493	LMNB1	-4.63	Lower expression	<1.00E-07	<1.00E-07	0.039	CELL_CYCLE
A_23_P67725	LMNB2	-4.26	Lower expression	5.91E-06	1.19E-04	0.058	
A_23_P372074	LOC100294362	-4.82	Lower expression	<1.00E-07	<1.00E-07	0.032	
A_24_P371738	LOC100506548	-3.87	Lower expression	6.70E-05	9.37E-04	0.084	
A_24_P734060	LOC284454	3.92	Higher expression	5.32E-05	7.84E-04	0.08	
A_33_P3283601	LOC389033	4.91	Higher expression	<1.00E-07	<1.00E-07	0.029	
A_24_P66932	LOC729080	-4.19	Lower expression	9.85E-06	1.86E-04	0.062	
A_23_P200222	LRP8	-4.52	Lower expression	3.94E-06	9.39E-05	0.043	
A_32_P98979	LRP8	-4.47	Lower expression	3.94E-06	9.39E-05	0.046	
A_33_P3253707	LRR1	-4.78	Lower expression	<1.00E-07	<1.00E-07	0.033	
A_24_P240259	LRRC31	4.48	Higher expression	3.94E-06	9.39E-05	0.045	
A_24_P181585	LRRC59	-4.91	Lower expression	<1.00E-07	<1.00E-07	0.029	
A_24_P240187	LRRN1	5.75	Higher expression	<1.00E-07	<1.00E-07	0.014	
A_23_P323783	LSM14B	-3.88	Lower expression	6.11E-05	8.62E-04	0.084	
A_33_P3248265	LTB	-6.26	Lower expression	<1.00E-07	<1.00E-07	0.009	IMMUNE
A_24_P264790	LTBP3	3.87	Higher expression	7.09E-05	9.85E-04	0.085	

A_23_P166848	LTF	6.97	Higher expression	<1.00E-07	<1.00E-07	0.004	IMMUNE
A_24_P317762	LY6E	-5.61	Lower expression	<1.00E-07	<1.00E-07	0.016	IMMUNE
A_33_P3359047	LYPD6	6.48	Higher expression	<1.00E-07	<1.00E-07	0.007	
A_24_P42264	LYZ	-5.87	Lower expression	<1.00E-07	<1.00E-07	0.012	IMMUNE
A_23_P92441	MAD2L1	-6.71	Lower expression	<1.00E-07	<1.00E-07	0.005	
A_32_P220715	MAP1LC3B	-3.89	Lower expression	5.52E-05	7.98E-04	0.082	
A_23_P41796	MAP3K1	4.45	Higher expression	3.94E-06	9.39E-05	0.047	KRAS_SIGNALING
A_33_P3368139	MAP3K1	4.26	Higher expression	5.91E-06	1.19E-04	0.058	KRAS_SIGNALING
A_33_P3247205	MARC1	-4.01	Lower expression	3.74E-05	6.06E-04	0.073	
A_23_P338495	MARCH8	4.66	Higher expression	<1.00E-07	<1.00E-07	0.038	METABOLIC
A_24_P258051	MASTL	-4.59	Lower expression	<1.00E-07	<1.00E-07	0.04	CELL_CYCLE
A_24_P114255	MBOAT2	4.71	Higher expression	<1.00E-07	<1.00E-07	0.036	METABOLIC
A_33_P3288246	MCC	5.57	Higher expression	<1.00E-07	<1.00E-07	0.016	WNT_BETA_CATENIN_SIGNALING
A_23_P90612	MCM6	-4.48	Lower expression	3.94E-06	9.39E-05	0.045	CELL_CYCLE
A_33_P3258223	MCM7	-3.94	Lower expression	4.93E-05	7.43E-04	0.078	CELL_CYCLE
A_23_P68547	MCM8	-4.94	Lower expression	<1.00E-07	<1.00E-07	0.028	
A_23_P321261	ME2	-4.68	Lower expression	<1.00E-07	<1.00E-07	0.037	METABOLIC
A_23_P105227	ME3	4.45	Higher expression	3.94E-06	9.39E-05	0.048	
A_23_P116614	ME3	4.19	Higher expression	9.85E-06	1.86E-04	0.062	
A_24_P940666	MED1	-4.45	Lower expression	3.94E-06	9.39E-05	0.047	ESTROGEN_RESPONSE
A_33_P3281795	MGLL	-3.92	Lower expression	5.12E-05	7.64E-04	0.08	METABOLIC
A_23_P159956	MID2	-3.9	Lower expression	5.52E-05	7.98E-04	0.081	IMMUNE
A_33_P3229107	MIR205HG	5.22	Higher expression	<1.00E-07	<1.00E-07	0.02	
A_33_P3328360	MIRLET7BHG	5.82	Higher expression	<1.00E-07	<1.00E-07	0.013	
A_32_P330000	MIRLET7BHG	4.26	Higher expression	5.91E-06	1.19E-04	0.057	
A_23_P252335	MIS18A	-4.95	Lower expression	<1.00E-07	<1.00E-07	0.028	
A_23_P390068	MISP	-4.42	Lower expression	3.94E-06	9.39E-05	0.049	
A_33_P3374210	MKI67	-4.85	Lower expression	<1.00E-07	<1.00E-07	0.031	CELL_CYCLE
A_23_P13873	MLF2	-5.03	Lower expression	<1.00E-07	<1.00E-07	0.025	
A_23_P13872	MLF2	-4.11	Lower expression	1.97E-05	3.50E-04	0.066	
A_23_P40174	MMP9	-5.74	Lower expression	<1.00E-07	<1.00E-07	0.014	
A_32_P185317	MNX1-AS1	-4.49	Lower expression	3.94E-06	9.39E-05	0.045	
A_23_P73835	MOSPD1	-3.87	Lower expression	7.09E-05	9.85E-04	0.085	METABOLIC
A_24_P279797	MRI1	3.9	Higher expression	5.52E-05	7.98E-04	0.081	
A_23_P170352	MRPL12	-5.5	Lower expression	<1.00E-07	<1.00E-07	0.017	
A_23_P10346	MRPL12	-5.09	Lower expression	<1.00E-07	<1.00E-07	0.023	
A_33_P3365810	MRPL12	-4.62	Lower expression	<1.00E-07	<1.00E-07	0.039	
A_23_P106998	MRPS23	-4.53	Lower expression	3.94E-06	9.39E-05	0.043	
A_23_P392942	MSR1	-4.75	Lower expression	<1.00E-07	<1.00E-07	0.035	IMMUNE
A_24_P148796	MST1	4.64	Higher expression	<1.00E-07	<1.00E-07	0.038	COAGULATION
A_23_P252413	MT2A	-4.21	Lower expression	5.91E-06	1.19E-04	0.06	IMMUNE
A_23_P120316	MTHFD2	-4.74	Lower expression	<1.00E-07	<1.00E-07	0.035	
A_24_P55465	MTPN	-4.27	Lower expression	5.91E-06	1.19E-04	0.057	IMMUNE
A_23_P17663	MX1	-4.31	Lower expression	3.94E-06	9.39E-05	0.054	
A_23_P6263	MX2	-4.65	Lower expression	<1.00E-07	<1.00E-07	0.038	IMMUNE
A_24_P367227	MYBL1	-5.19	Lower expression	<1.00E-07	<1.00E-07	0.021	ESTROGEN_RESPONSE
A_23_P43157	MYBL1	-5.17	Lower expression	<1.00E-07	<1.00E-07	0.022	ESTROGEN_RESPONSE
A_33_P3346891	MYBL1	-4.51	Lower expression	3.94E-06	9.39E-05	0.044	ESTROGEN_RESPONSE

A_24_P915007	NACC1	-4.59	Lower expression	<1.00E-07	<1.00E-07	0.041	
A_23_P95594	NAT1	4.64	Higher expression	<1.00E-07	<1.00E-07	0.038	UV_RESPONSE
A_32_P171181	NBPF10	3.87	Higher expression	7.09E-05	9.85E-04	0.085	
A_33_P3368800	NBPF12	4.41	Higher expression	3.94E-06	9.39E-05	0.05	
A_23_P155815	NCAPG	-7.31	Lower expression	<1.00E-07	<1.00E-07	0.003	
A_33_P3230254	NCAPG	-4.15	Lower expression	1.38E-05	2.52E-04	0.065	
A_23_P120442	NCOA3	-4.75	Lower expression	<1.00E-07	<1.00E-07	0.035	IMMUNE
A_23_P313560	NCOA3	-4.08	Lower expression	2.56E-05	4.42E-04	0.068	IMMUNE
A_24_P185604	NDRG3	-3.99	Lower expression	4.14E-05	6.47E-04	0.075	
A_23_P122228	NDUFS6	-4.14	Lower expression	1.38E-05	2.52E-04	0.065	METABOLIC
A_23_P129157	NEIL1	5.11	Higher expression	<1.00E-07	<1.00E-07	0.023	DNA_REPAIR
A_33_P3422213	NEK10	6.24	Higher expression	<1.00E-07	<1.00E-07	0.009	CELL_CYCLE
A_33_P3284045	NEK10	5.63	Higher expression	<1.00E-07	<1.00E-07	0.016	CELL_CYCLE
A_23_P353574	NEK7	-3.87	Lower expression	6.70E-05	9.37E-04	0.085	METABOLIC
A_32_P155026	NFIA	4.11	Higher expression	1.97E-05	3.50E-04	0.067	
A_23_P85682	NFIA	4	Higher expression	3.94E-05	6.27E-04	0.075	
A_33_P3884230	NFIX	4.54	Higher expression	1.97E-06	5.50E-05	0.042	
A_33_P3281785	NKX3-1	7.59	Higher expression	<1.00E-07	<1.00E-07	0.002	HOMEOBOX
A_33_P3489737	NLN	-4.42	Lower expression	3.94E-06	9.39E-05	0.049	METABOLIC
A_24_P101402	NOP56	-4.27	Lower expression	5.91E-06	1.19E-04	0.057	
A_33_P3212645	NOTCH2NL	4.11	Higher expression	2.17E-05	3.81E-04	0.067	NOTCH_SIGNALING
A_23_P69699	NPY1R	4.33	Higher expression	3.94E-06	9.39E-05	0.053	ESTROGEN_RESPONSE
A_23_P206661	NQO1	-3.96	Lower expression	4.53E-05	6.95E-04	0.077	ESTROGEN_RESPONSE
A_32_P486693	NRIP3	6.78	Higher expression	<1.00E-07	<1.00E-07	0.005	
A_33_P3244956	NRIP3	8.85	Higher expression	<1.00E-07	<1.00E-07	<0.001	
A_33_P3368471	NT5DC1	4.87	Higher expression	<1.00E-07	<1.00E-07	0.031	
A_23_P204630	NTN4	6.78	Higher expression	<1.00E-07	<1.00E-07	0.005	
A_24_P359671	NTNG1	4.76	Higher expression	<1.00E-07	<1.00E-07	0.034	EPITHELIAL_STRUCTURE
A_33_P3350488	NUSAP1	-7.31	Lower expression	<1.00E-07	<1.00E-07	0.003	CELL_CYCLE
A_23_P118038	NUTF2	-4.48	Lower expression	3.94E-06	9.39E-05	0.046	
A_23_P64828	OAS1	-4.39	Lower expression	3.94E-06	9.39E-05	0.05	IMMUNE
A_23_P204087	OAS2	-4.08	Lower expression	2.56E-05	4.42E-04	0.069	IMMUNE
A_33_P3402489	OAS3	-4.52	Lower expression	3.94E-06	9.39E-05	0.043	IMMUNE
A_23_P139786	OASL	-4.71	Lower expression	<1.00E-07	<1.00E-07	0.036	IMMUNE
A_23_P399217	OR5P3	3.96	Higher expression	4.53E-05	6.95E-04	0.077	
A_23_P87500	ORMDL2	-5.2	Lower expression	<1.00E-07	<1.00E-07	0.021	
A_23_P38190	ORMDL3	-4.35	Lower expression	3.94E-06	9.39E-05	0.053	
A_23_P118462	OVCA2	3.95	Higher expression	4.53E-05	6.95E-04	0.077	
A_33_P3447304	OXR1	-3.97	Lower expression	4.53E-05	6.95E-04	0.077	APOPTOSIS
A_23_P116674	PA2G4	-4.45	Lower expression	3.94E-06	9.39E-05	0.047	
A_33_P3227209	PA2G4	-4.05	Lower expression	3.35E-05	5.54E-04	0.07	
A_23_P79942	PANK2	-4.14	Lower expression	1.38E-05	2.52E-04	0.065	METABOLIC
A_23_P397341	PAQR4	-7.69	Lower expression	<1.00E-07	<1.00E-07	0.001	
A_33_P3398448	PARP10	-4.49	Lower expression	3.94E-06	9.39E-05	0.045	
A_23_P111804	PARP12	-4.04	Lower expression	3.35E-05	5.54E-04	0.072	IMMUNE
A_23_P87769	PARPBP	-3.97	Lower expression	4.53E-05	6.95E-04	0.077	
A_33_P3225760	PCDH18	3.97	Higher expression	4.53E-05	6.95E-04	0.076	
A_33_P3393573	PCMT1	-4.35	Lower expression	3.94E-06	9.39E-05	0.053	

A_23_P28886	PCNA	-6.28	Lower expression	<1.00E-07	<1.00E-07	0.009	
A_33_P3420792	PDAP1	-4.77	Lower expression	<1.00E-07	<1.00E-07	0.034	PI3K_AKT_MTOR
A_23_P251095	PDHA1	-3.87	Lower expression	6.50E-05	9.13E-04	0.084	METABOLIC
A_23_P144796	PDLIM4	3.91	Higher expression	5.32E-05	7.84E-04	0.08	EPITHELIAL_MESENCHYMAL_TRANSITION
A_23_P21618	PDZRN3	4.16	Higher expression	9.85E-06	1.86E-04	0.064	
A_33_P3268334	PGAM1	4.35	Higher expression	3.94E-06	9.39E-05	0.053	METABOLIC
A_23_P125829	PGK1	-4.37	Lower expression	3.94E-06	9.39E-05	0.051	METABOLIC
A_23_P24555	PHLDB1	4.56	Higher expression	<1.00E-07	<1.00E-07	0.042	ADIPOGENESIS
A_23_P307844	PHYHD1	5.2	Higher expression	<1.00E-07	<1.00E-07	0.021	
A_24_P29401	PIK3R1	4.64	Higher expression	<1.00E-07	<1.00E-07	0.038	
A_23_P31224	PILRA	3.97	Higher expression	4.53E-05	6.95E-04	0.076	
A_23_P8702	PIP	6.03	Higher expression	<1.00E-07	<1.00E-07	0.011	APOPTOSIS
A_33_P3397443	PKMYT1	-7.06	Lower expression	<1.00E-07	<1.00E-07	0.004	CELL_CYCLE
A_24_P183128	PLAC8	-4.96	Lower expression	<1.00E-07	<1.00E-07	0.027	HYPOXIA
A_23_P339240	PLCH1	-4.27	Lower expression	5.91E-06	1.19E-04	0.057	
A_24_P334248	PLCH1	-3.9	Lower expression	5.52E-05	7.98E-04	0.081	
A_24_P313504	PLK1	-5.76	Lower expression	<1.00E-07	<1.00E-07	0.014	CELL_CYCLE
A_23_P57961	PLXNB1	4.23	Higher expression	5.91E-06	1.19E-04	0.06	ESTROGEN_RESPONSE
A_23_P140256	PNP	-4.98	Lower expression	<1.00E-07	<1.00E-07	0.027	
A_33_P3666884	PNPLA7	4	Higher expression	3.94E-05	6.27E-04	0.074	
A_23_P368154	PODN	4.37	Higher expression	3.94E-06	9.39E-05	0.051	
A_33_P3266780	PODXL2	-4.04	Lower expression	3.35E-05	5.54E-04	0.071	
A_23_P71146	POLD2	-4.43	Lower expression	3.94E-06	9.39E-05	0.048	
A_33_P3299834	POLR2E	-4.05	Lower expression	3.35E-05	5.54E-04	0.071	DNA_REPAIR
A_23_P157449	POLR2K	-5.22	Lower expression	<1.00E-07	<1.00E-07	0.02	DNA_REPAIR
A_23_P157452	POLR2K	-4.63	Lower expression	<1.00E-07	<1.00E-07	0.039	DNA_REPAIR
A_23_P31399	PON2	5.93	Higher expression	<1.00E-07	<1.00E-07	0.012	
A_33_P3350726	PPARG	4.41	Higher expression	3.94E-06	9.39E-05	0.049	METABOLIC
A_23_P302116	PPFIA1	-4.59	Lower expression	<1.00E-07	<1.00E-07	0.041	
A_32_P96272	PPIAL4A	-4.6	Lower expression	<1.00E-07	<1.00E-07	0.04	
A_24_P376309	PPP1R9B	-4.71	Lower expression	<1.00E-07	<1.00E-07	0.036	
A_23_P166360	PRAME	-4.66	Lower expression	<1.00E-07	<1.00E-07	0.038	APOPTOSIS
A_23_P206059	PRC1	-4.45	Lower expression	3.94E-06	9.39E-05	0.048	CELL_CYCLE
A_23_P11995	PRDX1	-4.31	Lower expression	3.94E-06	9.39E-05	0.055	REACTIVE_OXYGEN_SPECIES_PATHWAY
A_23_P114232	PRDX4	-5.96	Lower expression	<1.00E-07	<1.00E-07	0.012	
A_23_P983	PRDX6	-3.87	Lower expression	6.90E-05	9.61E-04	0.085	REACTIVE_OXYGEN_SPECIES_PATHWAY
A_24_P18146	PSD3	5.47	Higher expression	<1.00E-07	<1.00E-07	0.017	
A_33_P3226605	PSIP1	-3.89	Lower expression	5.52E-05	7.98E-04	0.082	CELL_CYCLE
A_23_P25735	PSMA6	-3.9	Lower expression	5.52E-05	7.98E-04	0.081	MYC_TARGETS
A_23_P91468	PSMA7	-5.68	Lower expression	<1.00E-07	<1.00E-07	0.015	MYC_TARGETS
A_23_P100576	PSMB3	-4.36	Lower expression	3.94E-06	9.39E-05	0.052	MYC_TARGETS
A_23_P258570	PSMD10	-3.99	Lower expression	4.14E-05	6.47E-04	0.075	
A_23_P77876	PSMD12	-4.98	Lower expression	<1.00E-07	<1.00E-07	0.026	PI3K_AKT_MTOR
A_32_P12639	PSMD12	-4.67	Lower expression	<1.00E-07	<1.00E-07	0.038	PI3K_AKT_MTOR
A_23_P112613	PSMD12	-3.92	Lower expression	5.32E-05	7.84E-04	0.08	PI3K_AKT_MTOR
A_23_P26783	PSMD3	-4.49	Lower expression	3.94E-06	9.39E-05	0.045	MYC_TARGETS
A_24_P289726	PSMD3	-4.26	Lower expression	5.91E-06	1.19E-04	0.058	MYC_TARGETS

A_23_P106741	PSMD7	-5.9	Lower expression	<1.00E-07	<1.00E-07	0.012	MYC_TARGETS
A_23_P68717	PSMG1	-4.35	Lower expression	3.94E-06	9.39E-05	0.053	PI3K_AKT_MTOR
A_24_P870620	PTN	4.32	Higher expression	3.94E-06	9.39E-05	0.054	ESTROGEN_RESPONSE
A_23_P105436	PTPN11	-4.18	Lower expression	9.85E-06	1.86E-04	0.062	PI3K_AKT_MTOR
A_33_P3241984	PTPN22	-4.87	Lower expression	<1.00E-07	<1.00E-07	0.03	PROTEIN_SECRETION
A_23_P125451	PTPRC	-4.32	Lower expression	3.94E-06	9.39E-05	0.054	
A_33_P3296482	PTPRK	4.06	Higher expression	3.15E-05	5.33E-04	0.069	
A_23_P135576	PTPRT	4.9	Higher expression	<1.00E-07	<1.00E-07	0.029	
A_23_P7636	PTTG1	-6.31	Lower expression	<1.00E-07	<1.00E-07	0.009	CELL_CYCLE
A_23_P60024	PTTG1	-5.06	Lower expression	<1.00E-07	<1.00E-07	0.024	CELL_CYCLE
A_23_P60016	PTTG3P	-4.26	Lower expression	5.91E-06	1.19E-04	0.058	CELL_CYCLE
A_23_P141894	PVR	-4.31	Lower expression	3.94E-06	9.39E-05	0.055	
A_23_P107612	RAB27B	4.32	Higher expression	3.94E-06	9.39E-05	0.054	
A_33_P3314594	RAB37	-4.1	Lower expression	2.17E-05	3.81E-04	0.067	
A_33_P3275998	RABEP1	5.28	Higher expression	<1.00E-07	<1.00E-07	0.019	ESTROGEN_RESPONSE
A_24_P945147	RABEP1	4.8	Higher expression	<1.00E-07	<1.00E-07	0.032	ESTROGEN_RESPONSE
A_23_P78158	RABEP1	4.37	Higher expression	3.94E-06	9.39E-05	0.051	ESTROGEN_RESPONSE
A_32_P186474	RACGAP1	-7.35	Lower expression	<1.00E-07	<1.00E-07	0.002	CELL_CYCLE
A_23_P65110	RACGAP1	-6.56	Lower expression	<1.00E-07	<1.00E-07	0.007	CELL_CYCLE
A_23_P65041	RACGAP1P	-7.48	Lower expression	<1.00E-07	<1.00E-07	0.002	
A_23_P20463	RAD21	-5.02	Lower expression	<1.00E-07	<1.00E-07	0.025	CELL_CYCLE
A_23_P74115	RAD54L	-4.01	Lower expression	3.74E-05	6.06E-04	0.073	CELL_CYCLE
A_33_P3443165	RAE1	-4.93	Lower expression	<1.00E-07	<1.00E-07	0.028	DNA_REPAIR
A_24_P47547	RAN	-5.08	Lower expression	<1.00E-07	<1.00E-07	0.024	CELL_CYCLE
A_32_P506600	RAN	-4.28	Lower expression	3.94E-06	9.39E-05	0.056	CELL_CYCLE
A_23_P152970	RAPGEFL1	-4.77	Lower expression	<1.00E-07	<1.00E-07	0.034	ESTROGEN_RESPONSE
A_33_P3421571	RAPH1	4.24	Higher expression	5.91E-06	1.19E-04	0.059	
A_24_P183994	RASEF	5.01	Higher expression	<1.00E-07	<1.00E-07	0.025	
A_33_P3283420	RBM12B	-4.4	Lower expression	3.94E-06	9.39E-05	0.05	
A_33_P3345504	RC3H2	-3.95	Lower expression	4.53E-05	6.95E-04	0.078	
A_23_P106433	RCCD1	-3.97	Lower expression	4.53E-05	6.95E-04	0.076	
A_32_P25050	RDH10	-4.38	Lower expression	3.94E-06	9.39E-05	0.051	
A_33_P3262789	REEP6	4.24	Higher expression	5.91E-06	1.19E-04	0.059	METABOLIC
A_33_P3267460	RFX3	4.79	Higher expression	<1.00E-07	<1.00E-07	0.033	PROTEIN_SECRETION
A_33_P3387616	RHPN1	-3.87	Lower expression	6.50E-05	9.13E-04	0.084	
A_24_P82957	RNF114	-4.06	Lower expression	3.15E-05	5.33E-04	0.07	
A_24_P306810	RNF213	-6.51	Lower expression	<1.00E-07	<1.00E-07	0.007	IMMUNE
A_23_P327140	RNF213	-3.95	Lower expression	4.53E-05	6.95E-04	0.078	IMMUNE
A_33_P3230103	RNFT2	-3.96	Lower expression	4.53E-05	6.95E-04	0.077	
A_24_P73738	RPL13	-4.01	Lower expression	3.94E-05	6.27E-04	0.073	
A_33_P3246163	RPL5	3.92	Higher expression	5.12E-05	7.64E-04	0.079	
A_24_P63262	RPN1	-4.7	Lower expression	<1.00E-07	<1.00E-07	0.037	PI3K_AKT_MTOR
A_33_P3240258	RPN2	-4.08	Lower expression	2.76E-05	4.72E-04	0.069	
A_33_P3323384	RRM2B	-4.34	Lower expression	3.94E-06	9.39E-05	0.053	DNA_REPAIR
A_24_P28722	RSAD2	-5.06	Lower expression	<1.00E-07	<1.00E-07	0.024	
A_24_P3804	SAPCD1	-4.05	Lower expression	3.35E-05	5.54E-04	0.071	
A_24_P100517	SAPCD2	-4.18	Lower expression	9.85E-06	1.86E-04	0.062	
A_33_P3286621	SCARNA16	-5.7	Lower expression	<1.00E-07	<1.00E-07	0.015	

A_32_P163858	SCD	-3.97	Lower expression	4.53E-05	6.95E-04	0.076	METABOLIC
A_33_P3230990	SCUBE1	4.76	Higher expression	<1.00E-07	<1.00E-07	0.034	EPITHELIAL_STRUCTURE
A_23_P105144	SCUBE2	11.37	Higher expression	<1.00E-07	<1.00E-07	<0.001	ESTROGEN_RESPONSE
A_33_P3326682	SDHC	-4.12	Lower expression	1.77E-05	3.18E-04	0.066	METABOLIC
A_23_P17811	SEC14L2	8.21	Higher expression	<1.00E-07	<1.00E-07	0.001	ESTROGEN_RESPONSE
A_33_P3286754	SEC14L2	4.45	Higher expression	3.94E-06	9.39E-05	0.048	ESTROGEN_RESPONSE
A_24_P10226	SEMA6D	4.09	Higher expression	2.36E-05	4.11E-04	0.068	
A_23_P250212	SGK223	6.44	Higher expression	<1.00E-07	<1.00E-07	0.008	
A_24_P92256	SGSM2	4.14	Higher expression	1.38E-05	2.52E-04	0.065	
A_23_P212475	SHISA5	-4.06	Lower expression	3.15E-05	5.33E-04	0.07	
A_23_P169629	SHMT2	-4.61	Lower expression	<1.00E-07	<1.00E-07	0.04	
A_23_P156185	SHROOM1	4.77	Higher expression	<1.00E-07	<1.00E-07	0.033	CELL_CYCLE
A_23_P404902	SHROOM1	4.68	Higher expression	<1.00E-07	<1.00E-07	0.037	CELL_CYCLE
A_23_P17481	SIGLEC1	-5.8	Lower expression	<1.00E-07	<1.00E-07	0.013	IMMUNE
A_23_P50146	SIGLEC15	4.3	Higher expression	3.94E-06	9.39E-05	0.055	
A_24_P329924	SIK3	4.4	Higher expression	3.94E-06	9.39E-05	0.05	
A_32_P204205	SIX4	-4.45	Lower expression	3.94E-06	9.39E-05	0.047	HOMEOBOX
A_24_P322354	SKA1	-5.82	Lower expression	<1.00E-07	<1.00E-07	0.013	
A_23_P340909	SKA3	-4.27	Lower expression	5.91E-06	1.19E-04	0.057	
A_23_P200138	SLAMF8	-6.07	Lower expression	<1.00E-07	<1.00E-07	0.011	
A_23_P158725	SLC16A3	-5.66	Lower expression	<1.00E-07	<1.00E-07	0.015	METABOLIC
A_23_P120776	SLC25A1	-4.23	Lower expression	5.91E-06	1.19E-04	0.059	METABOLIC
A_24_P405313	SLC25A12	6.89	Higher expression	<1.00E-07	<1.00E-07	0.005	METABOLIC
A_23_P142714	SLC25A12	5.22	Higher expression	<1.00E-07	<1.00E-07	0.02	METABOLIC
A_33_P3350074	SLC25A19	-4.5	Lower expression	3.94E-06	9.39E-05	0.044	METABOLIC
A_24_P942211	SLC35E2	5.09	Higher expression	<1.00E-07	<1.00E-07	0.023	
A_33_P3274696	SLC52A2	-4.83	Lower expression	<1.00E-07	<1.00E-07	0.031	
A_24_P15621	SLC6A10P	-6.07	Lower expression	<1.00E-07	<1.00E-07	0.011	
A_23_P159937	SLC6A8	-4.11	Lower expression	1.97E-05	3.50E-04	0.066	
A_24_P335620	SLC7A5	-6.01	Lower expression	<1.00E-07	<1.00E-07	0.011	ESTROGEN_RESPONSE
A_33_P3378835	SLC9A3R1	-4.42	Lower expression	3.94E-06	9.39E-05	0.049	PI3K_AKT_MTOR
A_23_P144348	SLIT2	4.23	Higher expression	5.91E-06	1.19E-04	0.06	
A_23_P58588	SLIT3	4.38	Higher expression	3.94E-06	9.39E-05	0.051	EPITHELIAL_MESENCHYMAL_TRANSITION
A_23_P252764	SMARCA2	4.59	Higher expression	<1.00E-07	<1.00E-07	0.041	
A_23_P66487	SMARCD2	-4.23	Lower expression	5.91E-06	1.19E-04	0.059	
A_33_P3248519	SMC4	-6.52	Lower expression	<1.00E-07	<1.00E-07	0.007	CELL_CYCLE
A_24_P305764	SMS	-6.18	Lower expression	<1.00E-07	<1.00E-07	0.01	METABOLIC
A_23_P21776	SNAPC5	-4.33	Lower expression	3.94E-06	9.39E-05	0.053	DNA_REPAIR
A_33_P3280930	SNHG6	-5.18	Lower expression	<1.00E-07	<1.00E-07	0.021	
A_23_P324873	SNORA70	-4.17	Lower expression	9.85E-06	1.86E-04	0.063	
A_23_P4902	SNRNP70	5.01	Higher expression	<1.00E-07	<1.00E-07	0.025	
A_24_P98109	SNX10	-4.3	Lower expression	3.94E-06	9.39E-05	0.055	ESTROGEN_RESPONSE
A_23_P414252	SNX8	-4.06	Lower expression	3.15E-05	5.33E-04	0.07	
A_33_P3414192	SORCS1	4.41	Higher expression	3.94E-06	9.39E-05	0.049	
A_23_P304110	SOWAHA	4.26	Higher expression	5.91E-06	1.19E-04	0.058	
A_23_P327483	SPATA6	7.5	Higher expression	<1.00E-07	<1.00E-07	0.002	SPERMATOGENESIS
A_33_P3239242	SPATA6	4.2	Higher expression	9.85E-06	1.86E-04	0.061	SPERMATOGENESIS
A_33_P3489646	SPI1	-3.87	Lower expression	6.90E-05	9.61E-04	0.085	IMMUNE

A_23_P128698	SPRY2	4.09	Higher expression	2.36E-05	4.11E-04	0.068	KRAS_SIGNALING
A_33_P3373560	SPTAN1	4.15	Higher expression	1.38E-05	2.52E-04	0.064	
A_32_P199551	SPTSSB	-4.08	Lower expression	2.56E-05	4.42E-04	0.068	
A_23_P146284	SQLE	-6.86	Lower expression	<1.00E-07	<1.00E-07	0.005	
A_23_P30223	SRD5A1	-4.47	Lower expression	3.94E-06	9.39E-05	0.046	PI3K_AKT_MTOR
A_23_P86653	SRGN	-4.58	Lower expression	<1.00E-07	<1.00E-07	0.041	IMMUNE
A_23_P377819	SRSF5	5.57	Higher expression	<1.00E-07	<1.00E-07	0.016	
A_33_P3366146	SRSF5	5.5	Higher expression	<1.00E-07	<1.00E-07	0.017	
A_23_P102060	SSFA2	4.57	Higher expression	<1.00E-07	<1.00E-07	0.041	
A_33_P3366758	ST8SIA6	5.52	Higher expression	<1.00E-07	<1.00E-07	0.017	
A_32_P152195	STAC2	4.17	Higher expression	9.85E-06	1.86E-04	0.063	
A_23_P342727	STARD13	4.54	Higher expression	3.94E-06	9.39E-05	0.043	ANGIOGENESIS
A_24_P274270	STAT1	-4.05	Lower expression	3.35E-05	5.54E-04	0.071	IMMUNE
A_23_P416395	STC2	4.51	Higher expression	3.94E-06	9.39E-05	0.044	ESTROGEN_RESPONSE
A_32_P85676	STK32B	6.79	Higher expression	<1.00E-07	<1.00E-07	0.005	
A_33_P3317523	STMN1	-6.12	Lower expression	<1.00E-07	<1.00E-07	0.01	CELL_CYCLE
A_23_P309837	STON2	4.49	Higher expression	3.94E-06	9.39E-05	0.045	
A_33_P3222783	SURF4	-4.49	Lower expression	3.94E-06	9.39E-05	0.045	
A_23_P401076	SUSD3	4.93	Higher expression	<1.00E-07	<1.00E-07	0.028	
A_33_P3322870	SYT9	5.13	Higher expression	<1.00E-07	<1.00E-07	0.022	PROTEIN_SECRETION
A_23_P212844	TACC3	-3.88	Lower expression	6.11E-05	8.62E-04	0.084	CELL_CYCLE
A_23_P149529	TACSTD2	4.83	Higher expression	<1.00E-07	<1.00E-07	0.031	
A_23_P59005	TAP1	-4.72	Lower expression	<1.00E-07	<1.00E-07	0.036	
A_24_P49106	TCEAL7	4.43	Higher expression	3.94E-06	9.39E-05	0.048	
A_24_P243881	TCEB1	-4.37	Lower expression	3.94E-06	9.39E-05	0.051	
A_24_P349965	TCF19	-4.2	Lower expression	9.85E-06	1.86E-04	0.061	CELL_CYCLE
A_24_P345822	TFG	-4.49	Lower expression	3.94E-06	9.39E-05	0.044	
A_23_P212617	TFRC	-6.18	Lower expression	<1.00E-07	<1.00E-07	0.01	
A_23_P200780	TGFBR3	4.4	Higher expression	3.94E-06	9.39E-05	0.05	EPITHELIAL_MESENCHYMAL_TRANSITION
A_32_P41405	THOC3	-5.36	Lower expression	<1.00E-07	<1.00E-07	0.019	
A_33_P3342056	TIGIT	-4.43	Lower expression	3.94E-06	9.39E-05	0.048	
A_23_P53276	TIMELESS	-6.03	Lower expression	<1.00E-07	<1.00E-07	0.011	CELL_CYCLE
A_33_P3380742	TIMM17B	-5.77	Lower expression	<1.00E-07	<1.00E-07	0.014	
A_23_P107421	TK1	-3.88	Lower expression	5.91E-05	8.40E-04	0.083	CELL_CYCLE
A_23_P502678	TM2D2	-4.17	Lower expression	9.85E-06	1.86E-04	0.063	
A_23_P15101	TMC5	4.01	Higher expression	3.74E-05	6.06E-04	0.073	
A_23_P157007	TMEM176B	-4.37	Lower expression	3.94E-06	9.39E-05	0.052	
A_23_P253677	TMEM192	-4.01	Lower expression	3.74E-05	6.06E-04	0.073	
A_23_P206369	TMEM208	-5.83	Lower expression	<1.00E-07	<1.00E-07	0.013	
A_23_P122805	TMEM209	-4.99	Lower expression	<1.00E-07	<1.00E-07	0.026	
A_24_P133017	TMEM25	4.89	Higher expression	<1.00E-07	<1.00E-07	0.03	
A_23_P203115	TMEM25	4.68	Higher expression	<1.00E-07	<1.00E-07	0.037	
A_32_P75264	TMEM26	5.22	Higher expression	<1.00E-07	<1.00E-07	0.02	
A_23_P382240	TMEM26	4.88	Higher expression	<1.00E-07	<1.00E-07	0.03	
A_33_P3251073	TMEM52	-4.53	Lower expression	3.94E-06	9.39E-05	0.043	
A_33_P3406661	TMEM63C	4.87	Higher expression	<1.00E-07	<1.00E-07	0.031	
A_33_P3494748	TMEM65	-4.09	Lower expression	2.36E-05	4.11E-04	0.068	
A_23_P325040	TMPO	-5.43	Lower expression	<1.00E-07	<1.00E-07	0.018	CELL_CYCLE

A_23_P30666	TNFRSF21	-3.92	Lower expression	5.32E-05	7.84E-04	0.08	IMMUNE
A_33_P3388618	TNK1	4.73	Higher expression	<1.00E-07	<1.00E-07	0.036	
A_23_P141180	TOM1L2	4.46	Higher expression	3.94E-06	9.39E-05	0.047	
A_24_P393838	TOMM20	-4.12	Lower expression	1.77E-05	3.18E-04	0.066	
A_23_P216355	TONSL	-5.57	Lower expression	<1.00E-07	<1.00E-07	0.016	DNA_REPAIR
A_23_P118834	TOP2A	-8.35	Lower expression	<1.00E-07	<1.00E-07	0.001	APOPTOSIS
A_33_P3264612	TPCN2	-4.55	Lower expression	1.97E-06	5.50E-05	0.042	
A_24_P166807	TPD52	-3.89	Lower expression	5.52E-05	7.98E-04	0.082	
A_24_P358328	TPI1P2	-5.46	Lower expression	<1.00E-07	<1.00E-07	0.018	
A_33_P3335966	TPM1	6.69	Higher expression	<1.00E-07	<1.00E-07	0.005	EPITHELIAL_MESENCHYMAL_TRANSITION
A_23_P206018	TPM1	5.08	Higher expression	<1.00E-07	<1.00E-07	0.024	EPITHELIAL_MESENCHYMAL_TRANSITION
A_24_P44462	TPM1	4.31	Higher expression	3.94E-06	9.39E-05	0.054	EPITHELIAL_MESENCHYMAL_TRANSITION
A_24_P393880	TPM3	-4.11	Lower expression	1.97E-05	3.50E-04	0.066	MYOGENESIS
A_23_P68610	TPX2	-7.65	Lower expression	<1.00E-07	<1.00E-07	0.002	CELL_CYCLE
A_33_P3406899	TRAK1	4.1	Higher expression	2.17E-05	3.81E-04	0.067	
A_24_P327050	TRAPPC2L	-4.16	Lower expression	9.85E-06	1.86E-04	0.063	
A_32_P396186	TRIM66	4.04	Higher expression	3.35E-05	5.54E-04	0.071	
A_33_P3339212	TRIP13	-6.24	Lower expression	<1.00E-07	<1.00E-07	0.009	CELL_CYCLE
A_33_P3407256	TRIP13	-4.89	Lower expression	<1.00E-07	<1.00E-07	0.03	CELL_CYCLE
A_23_P88753	TSR3	-4.01	Lower expression	3.94E-05	6.27E-04	0.074	
A_33_P3259615	TTC36	4.3	Higher expression	3.94E-06	9.39E-05	0.055	
A_23_P259586	TTK	-4.99	Lower expression	<1.00E-07	<1.00E-07	0.026	CELL_CYCLE
A_23_P128154	TUBA1C	4.75	Higher expression	<1.00E-07	<1.00E-07	0.034	
A_23_P154070	TUBA4A	6.55	Higher expression	<1.00E-07	<1.00E-07	0.007	CELL_CYCLE
A_24_P175519	TXN	-4.99	Lower expression	<1.00E-07	<1.00E-07	0.026	REACTIVE_OXYGEN_SPECIES_PATHWAY
A_23_P60248	TXN	-4.08	Lower expression	2.76E-05	4.72E-04	0.069	REACTIVE_OXYGEN_SPECIES_PATHWAY
A_33_P3351120	TXNRD1	-4.44	Lower expression	3.94E-06	9.39E-05	0.048	REACTIVE_OXYGEN_SPECIES_PATHWAY
A_23_P143207	UBE2C	-4.94	Lower expression	<1.00E-07	<1.00E-07	0.028	CELL_CYCLE
A_24_P297539	UBE2C	-9.19	Lower expression	<1.00E-07	<1.00E-07	<0.001	CELL_CYCLE
A_23_P39561	UBE2F	-3.9	Lower expression	5.52E-05	7.98E-04	0.081	DNA_REPAIR
A_23_P115482	UBE2T	-7.08	Lower expression	<1.00E-07	<1.00E-07	0.004	CELL_CYCLE
A_23_P487	UCK2	-4.15	Lower expression	1.38E-05	2.52E-04	0.064	
A_23_P17330	UCKL1	-5.07	Lower expression	<1.00E-07	<1.00E-07	0.024	
A_24_P180243	UGT2B28	4.67	Higher expression	<1.00E-07	<1.00E-07	0.038	METABOLIC
A_23_P136671	UGT2B7	6.4	Higher expression	<1.00E-07	<1.00E-07	0.008	
A_23_P208880	UHRF1	-5.03	Lower expression	<1.00E-07	<1.00E-07	0.025	DNA_REPAIR
A_33_P3236157	UNG	-4.76	Lower expression	<1.00E-07	<1.00E-07	0.034	
A_23_P127095	USMG5	-4.74	Lower expression	<1.00E-07	<1.00E-07	0.035	
A_32_P26330	USP10	-4.54	Lower expression	1.97E-06	5.50E-05	0.042	
A_23_P100196	USP10	-4.01	Lower expression	3.94E-05	6.27E-04	0.073	
A_24_P405190	USP33	4.49	Higher expression	3.94E-06	9.39E-05	0.045	
A_23_P130020	UTP18	-4.6	Lower expression	<1.00E-07	<1.00E-07	0.04	
A_23_P77590	VAC14	-4.75	Lower expression	<1.00E-07	<1.00E-07	0.034	
A_32_P163169	VDAC1	-4.6	Lower expression	<1.00E-07	<1.00E-07	0.04	
A_23_P145978	VIPR2	4.23	Higher expression	5.91E-06	1.19E-04	0.06	
A_24_P98251	VPS13B	-4.21	Lower expression	5.91E-06	1.19E-04	0.061	

A_24_P270769	VPS35	-3.9	Lower expression	5.52E-05	7.98E-04	0.082	
A_23_P518	VTCN1	4.06	Higher expression	3.15E-05	5.33E-04	0.07	
A_24_P109351	VWA8	4.15	Higher expression	9.85E-06	1.86E-04	0.064	
A_23_P65651	WARS	-4.23	Lower expression	5.91E-06	1.19E-04	0.06	
A_33_P3209651	WDFY4	-4.14	Lower expression	1.38E-05	2.52E-04	0.065	
A_24_P14932	WDR52	4.91	Higher expression	<1.00E-07	<1.00E-07	0.029	
A_33_P3254606	WDR62	-4.28	Lower expression	3.94E-06	9.39E-05	0.056	CELL_CYCLE
A_33_P3341499	WNT5A	4.93	Higher expression	<1.00E-07	<1.00E-07	0.028	EPITHELIAL_MESENCHYMAL_TRANSITION
A_33_P3258346	XAF1	-3.9	Lower expression	5.52E-05	7.98E-04	0.081	IMMUNE
A_23_P206822	XPO6	-4.19	Lower expression	9.85E-06	1.86E-04	0.062	
A_23_P120414	YWHAB	-3.89	Lower expression	5.52E-05	7.98E-04	0.082	PI3K_AKT_MTOR
A_24_P106681	YWHAG	-5.35	Lower expression	<1.00E-07	<1.00E-07	0.019	ADIPOGENESIS
A_32_P226149	YWHAZ	-6.67	Lower expression	<1.00E-07	<1.00E-07	0.006	UNFOLDED_PROTEIN_RESPONSE
A_24_P209571	YWHAZ	-4.79	Lower expression	<1.00E-07	<1.00E-07	0.033	UNFOLDED_PROTEIN_RESPONSE
A_23_P39682	ZAP70	-4.28	Lower expression	3.94E-06	9.39E-05	0.056	IMMUNE
A_33_P3423820	ZC3H3	-5.17	Lower expression	<1.00E-07	<1.00E-07	0.022	
A_24_P259276	ZDHHC24	-4.37	Lower expression	3.94E-06	9.39E-05	0.052	
A_32_P157385	ZFAS1	-3.93	Lower expression	5.12E-05	7.64E-04	0.079	
A_23_P253921	ZKSCAN7	5.54	Higher expression	<1.00E-07	<1.00E-07	0.017	
A_33_P3222728	ZKSCAN7	4	Higher expression	3.94E-05	6.27E-04	0.075	
A_24_P301629	ZNF155	4.06	Higher expression	3.15E-05	5.33E-04	0.07	
A_23_P412186	ZNF252P	-4.05	Lower expression	3.15E-05	5.33E-04	0.07	
A_23_P334857	ZNF385B	6.43	Higher expression	<1.00E-07	<1.00E-07	0.008	
A_33_P3397323	ZNF441	4.14	Higher expression	1.38E-05	2.52E-04	0.065	
A_23_P169978	ZNF608	4.96	Higher expression	<1.00E-07	<1.00E-07	0.027	
A_33_P3279241	ZNF608	4.62	Higher expression	<1.00E-07	<1.00E-07	0.039	
A_33_P3209025	ZNF623	-4	Lower expression	3.94E-05	6.27E-04	0.075	
A_33_P3209030	ZNF623	-3.92	Lower expression	5.12E-05	7.64E-04	0.079	
A_23_P78835	ZNF787	-3.87	Lower expression	6.11E-05	8.62E-04	0.084	
A_33_P3372285	ZNF81	3.93	Higher expression	5.12E-05	7.64E-04	0.079	
A_23_P55682	ZSCAN18	4.16	Higher expression	9.85E-06	1.86E-04	0.064	
A_24_P920880	ZSCAN18	3.93	Higher expression	5.12E-05	7.64E-04	0.079	
A_33_P3269723	ZSWIM7	3.91	Higher expression	5.32E-05	7.84E-04	0.08	DNA_REPAIR
A_23_P63789	ZWINT	-5.7	Lower expression	<1.00E-07	<1.00E-07	0.015	DNA_REPAIR

Supplementary Table S5. Result from Gene Set Enrichment Analysis (GSEA). List of Hallmark gene sets significantly (FDR<0.05) enriched of genes associated with the ultralow risk classification. ES = enrichment score, FDR = false discovery rate.

Gene Set	GSEA Result	ES	FDR
MYC_TARGETS	Down-regulated	-0.586	<0.001
CELL_CYCLE	Down-regulated	-0.439	<0.001
DNA_REPAIR	Down-regulated	-0.408	<0.001
UNFOLDED_PROTEIN_RESPONSE	Down-regulated	-0.424	<0.001
PI3K_AKT_MTOR	Down-regulated	-0.342	<0.001
IMMUNE	Down-regulated	-0.293	0.001
MYOGENESIS	Up-regulated	0.345	0.002
EPITHELIAL_MESENCHYMAL_TRANSITION	Up-regulated	0.347	0.003
METABOLIC	Down-regulated	-0.251	0.008
P53_PATHWAY	Down-regulated	-0.264	0.013
HEDGEHOG_SIGNALING	Up-regulated	0.415	0.022
APOPTOSIS	Down-regulated	-0.224	0.032
ESTROGEN_RESPONSE	Up-regulated	0.229	0.042

SUPPLEMENTARY REFERENCES

1. Rutqvist LE, Johansson H. Long-term follow-up of the randomized Stockholm trial on adjuvant tamoxifen among postmenopausal patients with early stage breast cancer. *Acta oncologica (Stockholm, Sweden).* 2007;46(2):133-145.
2. Parker JS, Mullins M, Cheang MC, et al. Supervised risk predictor of breast cancer based on intrinsic subtypes. *Journal of clinical oncology : official journal of the American Society of Clinical Oncology.* 2009;27(8):1160-1167.
3. Desmedt C, Haibe-Kains B, Wirapati P, et al. Biological processes associated with breast cancer clinical outcome depend on the molecular subtypes. *Clinical cancer research : an official journal of the American Association for Cancer Research.* 2008;14(16):5158-5165.
4. Ignatiadis M, Singhal SK, Desmedt C, et al. Gene modules and response to neoadjuvant chemotherapy in breast cancer subtypes: a pooled analysis. *Journal of clinical oncology : official journal of the American Society of Clinical Oncology.* 2012;30(16):1996-2004.
5. Tobin NP, Harrell JC, Lovrot J, et al. Molecular subtype and tumor characteristics of breast cancer metastases as assessed by gene expression significantly influence patient post-relapse survival. *Annals of oncology : official journal of the European Society for Medical Oncology.* 2015;26(1):81-88.
6. Bild AH, Yao G, Chang JT, et al. Oncogenic pathway signatures in human cancers as a guide to targeted therapies. *Nature.* 2006;439(7074):353-357.
7. Majumder PK, Febbo PG, Bikoff R, et al. mTOR inhibition reverses Akt-dependent prostate intraepithelial neoplasia through regulation of apoptotic and HIF-1-dependent pathways. *Nature medicine.* 2004;10(6):594-601.
8. Creighton CJ, Casa A, Lazard Z, et al. Insulin-like growth factor-I activates gene transcription programs strongly associated with poor breast cancer prognosis. *Journal of clinical oncology : official journal of the American Society of Clinical Oncology.* 2008;26(25):4078-4085.
9. Creighton CJ, Hilger AM, Murthy S, et al. Activation of mitogen-activated protein kinase in estrogen receptor alpha-positive breast cancer cells in vitro induces an in vivo molecular phenotype of estrogen receptor alpha-negative human breast tumors. *Cancer research.* 2006;66(7):3903-3911.

10. Loi S, Haibe-Kains B, Majjaj S, et al. PIK3CA mutations associated with gene signature of low mTORC1 signaling and better outcomes in estrogen receptor-positive breast cancer. *Proceedings of the National Academy of Sciences of the United States of America.* 2010;107(22):10208-10213.
11. Saal LH, Johansson P, Holm K, et al. Poor prognosis in carcinoma is associated with a gene expression signature of aberrant PTEN tumor suppressor pathway activity. *Proceedings of the National Academy of Sciences of the United States of America.* 2007;104(18):7564-7569.
12. Farmer P, Bonnefoi H, Anderle P, et al. A stroma-related gene signature predicts resistance to neoadjuvant chemotherapy in breast cancer. *Nature medicine.* 2009;15(1):68-74.
13. Teschendorff AE, Miremadi A, Pinder SE, et al. An immune response gene expression module identifies a good prognosis subtype in estrogen receptor negative breast cancer. *Genome biology.* 2007;8(8):R157.
14. Liberzon A, Birger C, Thorvaldsdottir H, et al. The Molecular Signatures Database (MSigDB) hallmark gene set collection. *Cell systems.* 2015;1(6):417-425.
15. The Gene Ontology Consortium. The Gene Ontology Resource: 20 years and still GOing strong. *Nucleic acids research.* 2019;47(D1):D330-d338.
